# A review of health effects associated with exposure to jet engine emissions in and around airports

**DOI:** 10.1186/s12940-020-00690-y

**Published:** 2021-02-06

**Authors:** Katja M. Bendtsen, Elizabeth Bengtsen, Anne T. Saber, Ulla Vogel

**Affiliations:** 1 National Research Centre for the Working Environment, Lersø Parkallé 105, DK-2100 Copenhagen, Denmark; 2 Department of Health Technology, Technical University of Denmark, DK-2800 Kgs Lyngby, Denmark

**Keywords:** Jet engine emissions, Airports, Occupational exposure, Particulate matter, Polycyclic aromatic hydrocarbons, Biomarkers

## Abstract

**Background:**

Airport personnel are at risk of occupational exposure to jet engine emissions, which similarly to diesel exhaust emissions include volatile organic compounds and particulate matter consisting of an inorganic carbon core with associated polycyclic aromatic hydrocarbons, and metals. Diesel exhaust is classified as carcinogenic and the particulate fraction has in itself been linked to several adverse health effects including cancer.

**Method:**

In this review, we summarize the available scientific literature covering human health effects of exposure to airport emissions, both in occupational settings and for residents living close to airports. We also report the findings from the limited scientific mechanistic studies of jet engine emissions in animal and cell models.

**Results:**

Jet engine emissions contain large amounts of nano-sized particles, which are particularly prone to reach the lower airways upon inhalation. Size of particles and emission levels depend on type of aircraft, engine conditions, and fuel type, as well as on operation modes. Exposure to jet engine emissions is reported to be associated with biomarkers of exposure as well as biomarkers of effect among airport personnel, especially in ground-support functions. Proximity to running jet engines or to the airport as such for residential areas is associated with increased exposure and with increased risk of disease, increased hospital admissions and self-reported lung symptoms.

**Conclusion:**

We conclude that though the literature is scarce and with low consistency in methods and measured biomarkers, there is evidence that jet engine emissions have physicochemical properties similar to diesel exhaust particles, and that exposure to jet engine emissions is associated with similar adverse health effects as exposure to diesel exhaust particles and other traffic emissions.

**Supplementary Information:**

The online version contains supplementary material available at 10.1186/s12940-020-00690-y.

## Background

**Fig. 1:**
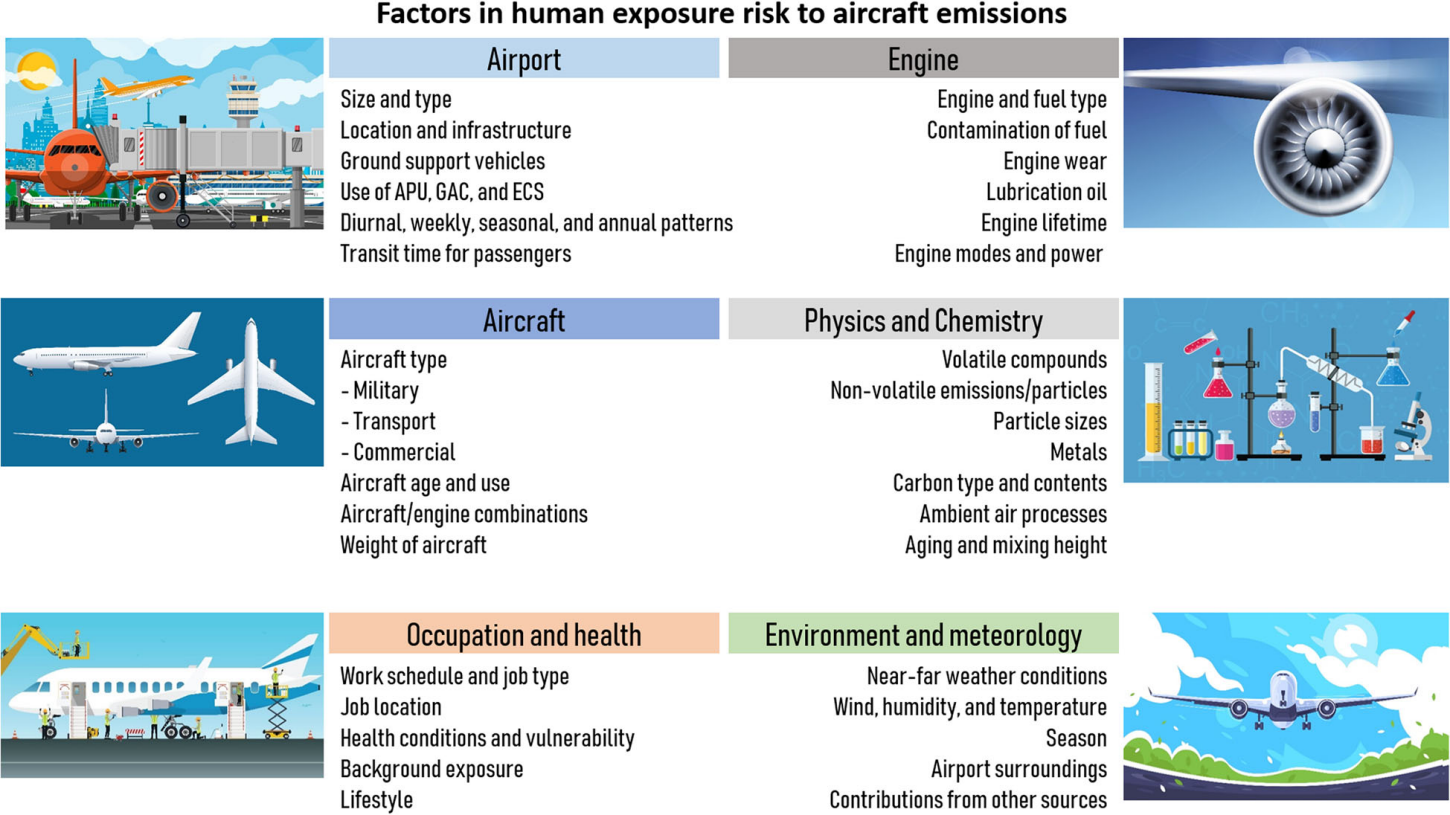
Overview of contributing factors in exposure risks from airports (APU: auxiliary power unit; GAC: ground air-conditioning cart, ECS: environmental control system).

Exposure to air pollution, including ultrafine particulate matter (UFP), from industry and traffic is associated with adverse health effects [1–4]. Airports are significant high-emission sources and human exposure to these emissions is a growing health concern. Importantly, airport personnel are at risk of occupational exposure to jet engine emissions [5]. More knowledge is needed on exposure risks, adverse health effects, biomarkers and risk management options related to the diverse factors influencing human exposure to airport emissions [6] (Fig. 1).

However, data collection seems challenging. Commercial airports are large, complex and diverse work places, where aircraft, ground-support equipment (GSE), and related vehicles all contribute to mixed emissions [7, 8]. In turn, commercial airports as well as military air stations are year-round active high security areas with restricted access, which can reduce the options for external researchers to collect optimal or sufficient measurements. Consensus or formal guidelines for optimal measurement design, instrumentation and analysis methods for the different emission components are lacking, which further complicates comparison of data and risk assessment [5, 9].

With this review, we seek to compile available studies in the open scientific literature on health effects of jet engine emissions in occupational settings and in residential areas around airports, along with mechanistic effects studied in animal and cell models. The studies were selected based on key papers and systematic searches (search terms, method and selection criteria are disclosed in the Additional file 1). We briefly summarize the characteristics of jet engine emissions and highlight the complexity of this field of research, but detailed research on emissions and physical-chemical studies is beyond the scope of this review.

## Toxicity of jet fuel exposure

The toxicity of (unburned) jet fuel as such has been considered in many studies (reviewed in [10]) since the early 1950’s, where the specifications of the hydrocarbon-based jet fuel, JP-4 (jet propellent-4), was published by the US air force. Major toxic effects reported for JP-4 were skin irritation, neurotoxicity, nephrotoxicity, and renal carcinogenicity in rats [11]. Jet fuels are mixtures of gasoline and kerosene with performance additives [10]. In 1994, US Air Force converted to JP-8, developed to be less volatile and less explosive upon crash incidents compared to JP-4. JP-8 (NATO F-34) is equivalent to Jet A-1 fuel used in commercial aircraft. A range of other kerosene-based jet fuels are in use, depending on aircraft type and differing in kerosene ratio and requirements for additives [5]. Measurements of a range of the common aircraft pollutants such as benzene, toluene, and chlorinated compounds in breath samples from exposed personnel on an airbase before and after work tasks showed significant exposure for all subjects, ranging from minor elevations up to > 100 times the values of the control group for fuel workers [12]. The uptake of JP-8 components both occur via inhalation and dermal contact, and apart from benzene, naphthalene in air and in exhaled breath condensate (EBC) may be useful as a biomarker of exposure to and uptake of JP-8 fuel components in the body [13]. Although most studies report low acute toxicity for both JP-4 and JP-8, JP-8 was reported to show effects such as respiratory tract sensory irritation [11], inflammatory cytokine secretion in exposed alveolar type II epithelial cells and in pulmonary alveolar macrophages [14], increased pulmonary resistance and decreased weight gain in rats upon inhalation exposure for 7 or 28 days [15, 16]. Subchronic 90-days studies with rats with various exposure levels of JP-4 and JP-8 showed little toxicity, apart from male rat hydrocarbon nephropathy [11]. However, JP-8 fuel exposure has been linked to noise-activated ototoxic hearing loss in animal studies [17, 18] and in occupational exposure cases [19, 20], and to immunotoxicity [21, 22].

It is likely that fuel refinements will advance in the future and be an important factor in emission reductions. A newer synthetic jet fuel (Fischer-Tropsch Synthetic Paraffinic Kerosene) under development to replace JP-8 in the future, was evaluated for toxicity in the required range of tests used to develop occupational exposure limits (OELs). The highest exposure level of 2000 mg/m^3^ (6 h per day, 5 days a week for 90 days) produced multifocal inflammatory cell infiltrations in rat lungs, whereas no genotoxicity or acute inhalation effects were observed, and the sensory irritation assay indicated that the refined synthetic fuel was less irritating than JP-8 [23]. Evidence of cancer risk is, however, normally evaluated in two-year inhalation studies in rats.

### Characteristics of jet engine emissions

Like other combustion engines, jet engines produce volatile organic compounds (VOC) such as CO_2_, NO_x_, CO, SO_x_ and low molecular weight polycyclic aromatic hydrocarbons (PAH), and particulate matter (PM) with associated PAH, and metals [24]. Incomplete combustion of fossil fuels, including kerosene, results in the formation of carbon-rich (> 60%), aromatic bi-products called char, and condensates, which are known as soot. Char and soot can either be measured as elemental carbon (EC, used in atmospheric sciences) or black carbon (BC, used in soil and sediment sciences) [25]. This terminology originates from their measurement methods (BC is light-absorbing, determined by optical methods and EC is refractory, determined by thermo-optical and oxidizing methods) [26]. BC is often used in physical/chemical aerosol studies of airport- and urban emissions, such as in Costabile et al. [27] and Keuken et al. [28]. However, there is no apparent consistent correlation between BC concentrations and particle number concentrations across exposure studies at airports, but data is limited as noted by Stacey [9].

In general, emission levels are high, but vary depending on engine conditions and fuel type, as well as on operation modes such as idling, taxi, take-off, climb-out and landing [29].

### Particulate matter (PM)

PM is divided by size ranges according to the aerodynamic diameter of the particles, where UFP are in the nanoscale of < 100 nm. Several studies have shown that aircraft emissions are dominated or even characterized by high concentrations of very small particles. This was underlined in a recent study by Stacey, Harrison and Pope carried out at Heathrow London in comparison to traffic background [30]. Some report particles in the range of 5–40 nm [31], and others particle diameters of 20 nm as compared to larger particles of > 35 nm measured at surrounding freeways [32]. Campagna et al. studied the contributions of UFP from a military airport to the surrounding area, by sampling on the airport grounds during flight activities, nearby the airport, in an urban area and in a rural area. The smallest primary particles were found within the airport (~ 10 nm) and the largest in the urban area (~ 72 nm). The highest UFP levels inside the airport were measured during taxi and take-off activities (4.0 × 10^6^ particles/cm^3^) [33]. Westerdahl et al. reported very high particle number concentrations at take-off of a single jet aircraft, with a 10 s peak of 4.8 million particles/cm^3^ together with elevated NO_x_ and BC levels [34].

The small particles are emitted in large numbers and tend to form complex agglomerates in ambient air that can be detected in larger particle size modes [35, 36] (see [5] for elaboration). In a recent study in Montreal-Pierre-Elliott-Trudeau International Airport, the total particle number concentration over all sizes at the airport apron reached 2.0 × 10^6^/cm^3^, which was significantly higher compared to downtown Montreal (1 × 10^4^/cm^3^). The geometric mean of observed ultrafine particle number density of nanoparticles was 1 × 10^5^/cm^3^ at the apron and 1.1 × 10^4^/cm^3^ outside the Departure Level entrance [37]. We recently published exposure measurements conducted at a commercial airport and non-commercial airfield, where air concentrations were measured to 7.7 × 10^6^ particle/cm^3^ or 1086 μg/m^3^ of total particles during take-off of one single jet plane [36]. The majority of these particles were below the size detection limit of 10 nm for the instruments [36], which was also shown, and highlighted as a general challenge, by others [38].

The nanostructure of carbon particles are influenced by fuel type and combustion processes. Low thrust settings are associated with the smallest particle sizes. In one of their studies, Vander Wal et al. characterized the aircraft particles as predominantly organic carbon at low thrust and EC at higher thrust settings [38]. In turn, it was reported that soot reactivity, characterized by an outer amorphous shell, of soot particles from a turbofan test engine was lower in particles from ground idle as compared to particles from climb-out engine mode for two fuel types. Biofuel blending slightly lowered this soot reactivity at ground idle, but had the opposite effect at the higher power condition of climb-out. The authors comment that for soot reactivity, measured by an outer amorphous shell in the study, biofuels may be beneficial in airports where ground idle engine conditions are often in use, but the effect on emissions in climb-out conditions is undetermined [31]. According Moore et al., a 50:50 biofuel blending reduces particle emissions from aircraft with 50–70%, compared to conventional Jet-A fuel [39]. Another study did extensive analyses of emissions from four on-wing commercial aircraft turbo engines (two newer CFM56–7 engines and two CFM56–3 engines), also demonstrating that the type of emissions were significantly dependent on power. PM emission indices (g/kg ^− 1^ fuel) were reported to increase from 0.011 to 0.205 g/kg ^− 1^ fuel with a power increase from idle to 85%. In turn, the data showed that hydrocarbons are mostly emitted at ground idle engine conditions, as opposed to PM emissions being more significant at higher power thrusts, such as take-off and landing. EC fraction of PM also increased with increase in power [40]. Targino et al. measured large EC (BC) concentrations during boarding and disembarking (mean 3.78 μg/m^3^), at the airport concourse (mean 3.16 μg/m^3^) and also inside an aircraft on the ground with open doors (mean 2.78 μg/m^3^) [41].

### Lubrication oil and organophosphate esters

A recent study found that intact forms of unburned jet engine lubrication oil was a major component of emissions from aircraft [42]. Organophosphate esters (OPEs) are a large group of chemicals with toxic properties used as stabilizing agents in numerous consumer – and industrial products, including in aircraft lubricating oil and hydraulic fluids. Airplane emissions are thought to be an important source of OPEs in the environment. Not only does these chemicals accumulate in ecosystems, but it is also a concern due to the location of airports near populated areas [5]. Li et al. recently studied the concentrations of 20 OPEs in ambient air, soil, pine needles, river water, and outdoor dust samples collected around an airport in Albany, New York, and reported elevated total OPE concentrations in all samples. The spatial distribution of OPEs in air, soil, and pine needles correlated with distance to the airport. The average daily intake of OPEs via air inhalation and outdoor dust ingestion in the vicinity of the airport was up to 1.53 ng/kg bw/day for children and 0.73 ng/kg bw/day for adults [43]. Another study examined organophosphates, such as tri-n-butyl phosphate, dibutyl phenyl phosphate, triphenyl phosphate and tricresyl phosphate from turbine and hydraulic oils, as well as oil aerosol/vapors and total volatile organic compounds (VOC) in air with potential for occupational exposure for airport ground personnel. The measured exposure levels were mainly below the limit of quantification during work tasks, but provoked exposure situations resulted in significantly higher exposure levels compared to normal conditions, illustrated by oil aerosol up to 240 mg/m^− 3^ and and tricresyl phosphate concentrations up to 31 mg/m^− 3^. Highest exposure levels were measured during loading from jet engine aircraft [44].

Exposure to toxic compounds via contaminated bleed air (from engine compressors), including OPEs, has been widely studied among cabin crew and pilots, and has been associated with adverse neurological effects and respiratory illness [45, 46].

### Metals and other elements

Metals which might be specific to airport emissions, either by abundance or type, such as the heavy-metal vanadium [47], could be potential chemical fingerprints. Abegglen et al. applied single particle mass spectrometry to investigate metal content and sources in emissions from different jet engines at various combustion conditions, and Mo, Ca, Na, Fe, Cu, Ba, Cr, Al, Si, Mg, Co, Mn, V, Ni, Pb, Ti and Zr were found to be significant frequently occurring metals. Fuel, lubrication oil, grease and engine wear are potential sources, but several metals were allocated to multiple sources [48].

In the studies of He et al and Shirmohammadi et al, particles were collected at Los Angeles Airport (LAX) and central Los Angeles (LA) and among other analyses, allocated according to elements associated with different sources [49, 50]. S was considered as aviation-related and particle-bound Na was viewed as ocean-related, due to sea salt from the ocean near by LAX. Al, Ca, Ti and K were considered as trace elements for road dust from LAX and central LA. Mn, Fe, Cu, Zn, Ba, Pb, Ni, and Mg were associated with traffic emissions, including fuel and lubricating oil combustions and brake abrasions, engine and tire wear. In LAX particles, S accounted for the largest fraction (49.5%), followed by road dust elements (21.8%) and traffic-related elements (15.9%). In particles from central LA, elements from traffic, road dust, and aviation were represented equally (28.5, 31.5, and 33.4%, respectively) [49, 50]. In a study from Montreal-Pierre-Elliott-Trudeau International Airport, several metals were found to be abundant in the particle fraction, such as Fe, Zn, and Al, and the authors speculate, that airports in fact may be hotspots for nanoparticles containing emerging contaminants [37]. A recent study investigated the levels of 57 elements at five sampling sites within the vicinity of Eskisehir Hasan Polatkan Airport in Turkey, based on moss bag biomonitoring using Sphagnum sp. in combination with chemical analyses of lubrication oil and aviation gasoline fuel used by general aviation, piston-engine, and turboprop aircraft. Moss bag biomonitoring was a useful tool in identification of the elements that accumulated downwind of the airport emissions. Characterization of the metal contents in moss bags and oil and fuel were in agreement, showing that Pb, along with Cd, Cu, Mo, Cr, Ni, Fe, Si, Zn, Na, P, Ca, Mg, and Al were dominating elements in the general aviation aircraft emissions [51].

### Polycyclic aromatic hydrocarbons/volatile organic compounds

Polycyclic aromatic hydrocarbons (PAH), including several known carcinogens, are also candidates for chemical airport emission tracers. PAH are semi-volatile compounds, in between the gaseous and particulate phases. Lighter-weight PAHs (< 4 rings) present almost exclusively in the vapour-phase and PAHs with higher molecular weights (> 4 rings) are almost completely particle-bound [5]. It was reported that the apron of the Fiumicino Airport in Rome had higher levels of measured PAH (27.2 μg/m^3^) compared to PAH levels in the airport building and terminal [52]. Another study of PAH in airport emissions at the apron reported that the five most abundant species of particle bound-PAHs for all sampling days were naphthalene, phenanthrene, fluoranthene, acenaphthene, and pyrene, with total concentrations between 0.152 μg/m^3^ - 0.189 μg/m^3^ (152.21–188.94 ng/m^3^) depending on season. The most abundant fractions of benzo(a)pyrene (BaP) equivalent concentration (BaPeq) in different molecular weights were high-weight PAHs (79.29%), followed by medium-weight PAHs (11.57%) and low-weight PAHs (9.14%). The percentages of total BaPeq in the very small particles < 0.032 μm were 52.4% (mean concentration 0.94 ng/m^3^) and 70.15% in particles < 100 μm (mean concentration 1.25 ng/m^3^) [53]. Studies of the emissions from a helicopter engine at different thrusts included analysis of 22 PAH compounds, where 97.5% of the total PAH emissions were two- and three-ringed PAHs, with a mean total PAH concentration of 843 μg/m^3^ and a maximum of 1653 μg/m^3^ during ground idle. This was 1.05–51.7 times higher compared to a heavy-duty diesel engine, a motor vehicle engine, and an F101 aircraft engine. In turn, total level of BaP during one landing and take-off cycle (LTO) (2.19 mg/LTO) [54] was higher than the European Commission emission factor of 1.24 mg/LTO, stated in their PAH position paper, where emission factors are used to calculate the degree to which a source contributes to the total emission of a specific pollutant [55]. The Danish occupational exposure limit for PAH is 200 μg/m^3^ [56], and reported PAH concentrations in ambient air across studies were below this level.

Volatile organic compounds (VOC) comprise a diverse group of organic chemicals, with different physicochemical and toxicological properties. Scientific studies of these emission compounds were meticulously reviewed by Masiol et al. [5], and as noted by the authors there is insufficient knowledge in terms of the significance of these compounds for airport exhaust health impacts [5]. Some VOC have known toxicities and other are suspected to have adverse health effects, and among the hydrocarbons found in aircraft exhaust, 14 single or complex compounds are listed as hazardous by the Federal Aviation Administration, which in addition to PAH compounds comprise benzene, styrene, xylene, toluene, acetaldehyde, 1,3-butadiene, n-hexane, acrolein, propionaldehyde, ethylbenzene, formaldehyde, and lead compounds [57]. A recent study assessed 46 VOC in the indoor air of the control tower maintenance room, potentially affecting employees, where a correlation was found between aircraft number and concentrations of light aldehydes/ketones [58].

### Summary and perspectives

Emission measurement studies are continuously conducted at international airports, such as Amsterdam Airport Schiphol (AMS) [28, 59], Rome Ciampino (CIA) [60], London Heathrow (LHR) [61, 62], Beirut-Rafic Hariri International Airport (RHIA) [63], Hartsfield-Jackson Atlanta International Airport [64], Los Angeles International Airport (LAX) [32, 49, 65], and other large airports in California [66] which besides measurements of the previously mentioned compounds, also often include analyses of emission patterns and weather conditions, and characterizations of particle size- and mass distributions [67]. The data from these emission studies and physical-chemical studies of emissions including particle matter (PM), from which we referenced some in the previous sections, were recently reviewed thoroughly [9]. To summarize the previous section, we repeat some selected important points regarding airport-sourced particles that were deducted from the available data by Stacey [9]:

1. *Particle numbers near airports are significantly higher than away from airports and jet engines are a significant source of UFP*. This means that urban areas in the vicinity of airports are at risk of increased exposure to UFP in addition to normal daily background and traffic-related emissions, but airport personnel working on the ground are in significant risk of exposure, simply due to proximity.
2. *The highest concentrations of UFP are measured downwind of aircraft*. Due to the occupational potential of exposure for airport ground workers there is a growing necessity of further studies of dispersion, size distributions and environmental factors affecting these emissions. Stacey [9] highlights that measurements at longer distances are highly influenced by physical and chemical processes affecting the emissions in the air, including volatile compounds. As such, there is a need for increased standardization of methods and instruments to facilitate valid comparisons between studies within this field, as has been established in general for environmental particulate matter (PM) measurements.
3. *Aircraft emissions are dominated by very small particles of < 20 nm.* This may be a way to separate these from other emission sources, such as road traffic, where the main particle fraction are of larger sizes. Smaller particle size means higher specific surface area. Smaller particles deposit in the deep end of the lung during inhalation and the total surface area of the deposited nanoparticles has been suggested to be predictive of toxicological potential in the lung [68].
4. *The majority of non-volatile airport emission particles are carbonaceous (consisting of elemental and organic carbon compounds)*.The emissions from aircraft consists of high numbers of soot particles with associated PAHs and metals, and thus, their physico-chemical composition is similar to diesel exhaust particles [36].

Diesel exhaust is classified as carcinogenic to humans by IARC [69], and cause lung cancer, systemic inflammation, and inflammatory responses in the airways [70]. Animal studies have shown that the particulate fraction of diesel exhaust is mutagenic and carcinogenic [71], whereas filtered diesel exhaust does not cause cancer [72]. Exposure to standard reference diesel particle SRM1650b and carbon black (CB) induce pulmonary acute phase response, neutrophil influx, and genotoxicity in mouse models [73–78]. Genotoxicity has been observed even at very low doses of CB [79]. In a meta-analysis of exposure to diesel exhaust and lung cancer occurrence in three occupational studies, the identified dose-response relationship showed that occupational exposure to 1 μg EC/m^3^ during a 45 year work life would cause 17 excess lung cancers per 10,000 exposed using the EC content of diesel exhaust as metric [80]. Another recent analysis of 14 case-control studies estimated exposure to diesel exhaust particles using job-exposure matrices. In this study, occupational exposure to 1 μg EC/m^3^ during a 45 year work life would cause 4 excess lung cancers per 10,000 exposed using the EC content of diesel exhaust as metric [81].

Carcinogenic substances are evaluated and listed by the International Agency of Research in Cancer (IARC) under WHO according to accumulated scientific findings in cellular, animal and human studies. Group 1 entails substances with sufficient evidence of carcinogenicity in humans and group 2 includes substances that IARC has classified as probably (2A) or possibly (2B) carcinogenic to humans [82]. As almost all current aviation fuel/jet fuels are extracted from the middle distillates of crude oil (kerosene fraction), which is between the fractions for gasoline and diesel [5] (whose combustion emissions are classified as group 2B and group 1 carcinogens, respectively [69]), there is cause for concern in terms of the potential carcinogenicity of exposure to jet fuel combustion products.

## Exposure studies

Reported exposure levels for PAH, BC and UPF in the studies below are presented in Table 1.

**Table 1:** Overview of reported levels of occupational exposures of PAH, BC, and particles in airports. *Mean levels are presented if reported. For detailed data, see references*

Description	***Reported mean levels*** ***Ambient air***	***Reported mean levels*** ***Personal monitors***	Reference
**PAH**			
Total mean PAH concentrations in integrated air samples at an airbase on different locations and in different flight-related and ground-support activities	601.1 ng/m^3^ (hangar background) 1025.4 ng/m^3^ (hangar taxiing) 2802.7 ng/m^3^ (engine test) 6795.3 ng/m^3^ (engine running on/off) 9811.1 ng/m^3^ (diesel-fueled aerospace ground equipment) *During flight-related exercises, PAH concentrations were 10–15 times higher than in ambient air*	*NA*	Childers et al. (2000) [1]
PAH compounds of highest levels measured for 24 h in three different locations	130–13,050 ng/m^3^ (naphthalene) 64–28,500 ng/m^3^ (2-methylnaphthalene) 24–35,300 ng/m^3^ (1-methylnaphtalene) 24–1610 ng/m^3^ (biphenyl) 54.2 ng/ m^3^ (fluoranthene) 8.6 ng/m^3^ (benzo[a]pyrene)	*NA*	Iavicoli et al. (2006) [2]
Total mean of 23 PAH (vapor and particle-bound) measured during 24 h of 5 work days at the airport apron, airport building and terminal/office area	27.703 μg/m^3^ (apron) 17.275 μg/m^3^ (airport building) 9.494 μg/m^3^ (terminal departure area) *Highest levels in the airport apron particularly for 1 and 2-methylnaphthalene and acenaphthene*	*NA*	Cavallo et al. (2006) [3]
Total mean particle-bound PAH measured in the vicinity of LAX to assess the spread of airport emissions in up – and downwind ambient air to the immediate neighborhood	18.2 ng/m^3^ (upwind from the airport) 24.6 ng/m^3^ (downwind from the airport) 50.1 ng/m^3^ (at the taxiway) 60.1 ng/m^3^ (terminal region) *Particle-bound PAH mean levels measured on two freeways were 47.0 ng/m*^*3*^ *and 169.4 ng/m*^*3*^	*NA*	Westerdahl et al. (2008) [4]
**Black carbon**			
Mean black carbon concentrations measured at different micro-environments of airports and in commercial flights	3.78 μg/m^3^ (during boarding/disembarking) 3.16 μg/m^3^ (airport concourse) 2.78 μg/m^3^ (inside aircraft with open doors) 0.81 μg/m^3^ (inside aircraft on the ground with closed doors)	*NA*	Targino et al. (2017) [5]
BC levels measured in the vicinity of LAX to assess the spread of airport emissions in up – and downwind ambient air to the immediate neighborhood	0.3 μg/cm^3^ (upwind from the airport) 0.7 μg/cm^3^ (downwind from the airport) 1.8 μg/cm^3^ (at the taxiway) 3.8 μg/cm^3^ (terminal region)	*NA*	Westerdahl et al. (2008) [4]
Contributions of airport activities to measured BC levels at Amsterdam Schiphol were measured for 32 sampling days over 6 months	Mean BC: 0.6 mg/m^3^	*NA*	Pirhadi et al. (2020) [6]
**Particles**			
UFP and size distributions measured in the vicinity of LAX to assess the spread of airport emissions in up – and downwind ambient air to the immediate neighborhood	Average UFP counts of 5 × 10^4^ particles/cm^3^ (500 m downwind of the airport), which were significantly influenced by aircraft operations where peaks were observed Maximum UFP measured was 4.8 × 10^6^ particles/m^3^ downwind from a jet aircraft taking off Particle size: 90 nm (upwind from airport) 10–15 nm (downwind from airport)	*NA*	Westerdahl et al. (2008) [4]
Total mean concentration of 10 daily UFP samples with personal monitors placed with crew chief and hangar operator	6.5 × 10^3^ particles/cm^3^ (downwind site)	2.5 × 10^4^ particles/cm^3^ (crew chief) 1.7 × 10^4^ particles/cm^3^ (hangar operator) *Median number concentrations for 2 months measurement period*	Buonanno et al. (2012) [7]
Geometric means of personal exposure to particle number concentration carried out in five different occupational groups	*NA*	37 × 10^3^ UFP/cm^3^ (baggage handlers) 5 × 10^3^ UFP/cm^3^ (landside security) 12–20 × 10^3^ UFP/cm^3^ (catering drivers, cleaning staff and airside security)	Møller et al. (2014) [8]
Particle and metal exposure in ambient air and in airport workers using exhaled breath condensates	1.0 × 10^4^–2.1 × 10^7^ particles/cm^3^ (apron workers) 10^3^–10^4^ (office staff) *Airport workers were exposed to significantly smaller particles (mean geometric size: 17.7 nm) compared to office workers (mean geometric size: 23.7 nm).*	Particulate content was found in exhaled breath condensates, but no difference was found between the two study groups	Marie-Desvergne et al. (2016) [9]
Number concentrations and size distributions inside the cabin of an aircraft waiting for take-off compared to outdoor	10–40 × 10^3^ particles/cm^3^ *A 40 min wait 100 m downwind of the runway was calculated to be equal to 4 h exposure in a clean urban background environment away from the airport*	*NA*	Ren et al. (2018)_a_ [10]
Potential exposure to passengers and indoor airport staff investigated by PM_2.5_ concentrations in the terminal building at three seasons	*Arrival hall:* 337 μg/m^3^ (Winter) 105 μg/m^3^ (Spring) 167 μg/m^3^ (Summer) *Departure hall:* 385 μg/m^3^ (Winter) 130 μg/m^3^ (Spring) 170 μg/m^3^ (Summer) *Ambient airport air:* 400 μg/m^3^ (Winter) 156 μg/m^3^ (Spring) 216 μg/m^3^ (Summer) *1.9–5.9 times higher particles number concentrations in the terminal buildings than measured in a normal urban environment* *Total UFP exposure during an entire average waiting period (including in the terminal building and airliner cabin) of a passenger was estimated to be equivalent to 11 h of exposure to normal urban emissions*	*NA*	Ren et al. (2018)_b_ [11]
UFP monitoring at several sampling sites in the vicinity of Lisbon Airport for 19 non-consecutive days	Downwind average particle number concentration range: 3.3 × 10^4^ cm^3^ to 5.9 × 10^4^ particles per cm^3^ Measured range of peaks: 2.3 × 10^5^ particles per cm^3^ to 3.4 × 10^5^ particles per cm^3^	*NA*	Lopes et al. (2019) [12]
Maximal measurements at a commercial airport and exposure assessment at a non-commercial airfield	10^6^ -10^8^ particles/cm^3^ (main combustion events of plane leaving and arriving) 1086 μg/m^3^ (single peak event of plane leaving) *10.7% was predicted to deposit in the alveolar lung regions*	*Personal exposure levels were similar to air concentrations*	Bendtsen et al. (2019) [13]
Maximal UFP number concentration of UFP exposures investigated for 33 male employees working in an airport taxiway	9.59 × 10^6^ (during support tasks in taxiing and taking off of the aircraft)	2.44 × 10^3^ particles/cm^3^ *Median UFP number concentration*	Marcias et al. (2019) [14]
Contributions of airport activities to measured particle number concentrations (PNCs) at Amsterdam Schiphol were measured for 32 sampling days over 6 months	Mean total PNC: 35,308 particles/cm^3^ *Aircraft departures and aircraft arrivals contributed to 46.1 and 26.7% of PNC, respectively. Ground support equipment and local road traffic accounted for 6.5% of PNC and were characterized by diameters of 60–80 nm. Traffic from surrounding freeways was characterized by particles of 30–40 nm and contributed to 18% of PNC* Mean PM_2.5_: 7.4 mg/m^3^ Particle size range: 10–20 nm	NA	Pirhadi et al. (2020) [6]

References 1. Childers JW et al. Environmental health perspectives 2000, 108(9):853-862 [83]; 2. Iavicoli I et al. Journal of occupational and environmental medicine 2006, 48(8):815-822 [84]; 3. Cavallo D et al. Toxicology 2006, 223(1-2):26-35 [52]; 4. Westerdahl D et al. Atmospheric Environment 2008, 42(13):3143-3155 [34]; 5. Targino AC et al. Transportation Research Part D: Transport and Environment 2017, 52:128-138 [41]; 6. Pirhadi M et al. Environmental Pollution 2020, 260:114027 [85]; 7. Buonanno G et al. Environmental Pollution 2012, 170:78-87 [86]; 8. Møller KL et al. PLOS ONE 2014, 9(9):e106671 [87]; 9. Marie-Desvergne C et al. Journal of breath research 2016, 10(3):036006 [88]; 10. Ren J et al. Indoor and Built Environment 2017, 27(9):1247-1258 [89]; 11. Ren J et al. Atmospheric Environment 2018, 179:222-226 [90]; 12. Lopes M et al. Atmospheric Pollution Research 2019, 10(5):1454-1463 [91]; 13. Bendtsen KM et al. Particle and Fibre Toxicology 2019, 16(1):23 [36]; 14. Marcias G et al. 2019, Environments 6(3):35 [92]

### Occupational exposure

Childers et al. (2000): An extensive study of PAH concentrations at an airbase was carried out, using real-time monitors and air samplers on different locations and in different flight-related and ground-support activities. Airborne and particle-bound PAH were measured in a break room, downwind from an aircraft (C-130H) during engine tests, in a maintenance hangar, in an aircraft (C-130H) cargo bay during cargo-drop training and during engine running on/off loading and backup exercises, and downwind from aerospace ground equipment (diesel-powered electrical generator and a diesel-powered heater). Measurements were carried out with three different monitors. Total PAH concentrations followed a general trend of downwind from two diesel aerospace ground equipment units > engine on/off-loading exercise > engine tests > maintenance hangar during taxi and takeoff > background measurements in the maintenance hangar. Reported mean total PAH concentrations in integrated air samples (vapor phase) were 0.6011 μg/m^3^ (hangar background), 1.0254 μg/m^3^ (hangar taxiing), 2.8027 μg/m^3^ (engine test), 6.7953 μg/m^3^ (engine running on/off) and 9.8111 μg/m^3^ (aerospace ground equipment). Dominating PAH in all exposure scenario was naphthalene, the alkyl-substituted naphthalenes, and other PAHs in the vapor phase. Particle-bound PAHs, such as fluoranthene, pyrene, and benzo[a]pyrene were also found. During flight-related exercises, PAH concentrations were 10–15 higher than in ambient air, and it was found that PAH contents fluctuated rapidly from < 0.02 to > 4 μg/m^3^ during flight-related activities [83].

Iavicoli et al. (2006): In this study, occupational exposure risk to PAH and biphenyl was evaluated in an Italian airport during winter. Concentration and purification of 12 samples of 25 PAH by gas chromatography-ion trap mass spectrometry sampled for 24 h in three different locations of the airport showed general low levels, with highest levels of naphthalene (0.13–13.05 μg/m^3^), 2-methylnaphthalene (0.064–28.5 μg/m^3^), 1-methylnaphtalene (0.024–35.3 μg/m^3^), and biphenyl (0.024–1.610 μg/m^3^). Measured levels of the carcinogens benzo[b + j + k]fluoranthene and benzo[a]pyrene were 0.0542 μg/m^3^ and 0.0086 μg/m^3^ respectively [84].

Buonanno et al. (2012): Occupational exposure and particle number distributions were studied at an aviation base on a downwind site, close to the airstrip and by 10 daily UFP samples with personal monitors placed with a crew chief (assists the pilots during ground activities) and a hangar operator (aircraft maintenance). Particle number distribution averaged a total concentration of 6.5 × 10^3^ particles/cm^3^ at the downwind site. Short-term peaks during the working day mainly related to takeoff, landing and pre-flight operations of jet engines were measured in the proximity of the airstrip. Personal exposure concentrations were higher than stationary monitoring measurements. Personal exposure of workers were at a median number concentration of 2.5 × 10^4^ particles/cm^3^ for the crew chief and 1.7 × 10^4^ particles/cm^3^ for the hangar operator during the 2 months measurement period. The crew chief experienced the highest exposures, with maximum values at approximately 8 × 10^4^ particles/cm^3^ [86].

Møller et al. (2014): Personal exposure monitoring of particle number concentration was carried out in five different occupational groups, namely baggage handlers, catering drivers, cleaning staff, airside security and landside security in CPH, for 8 days distributed over 2 weeks. The study reported significant differences among the occupational groups. Highest exposures were found in baggage handlers (geometric mean: 37 × 10^3^ UFP/cm^3^), which was 7 times higher in average compared to landside security which are indoor employees (geometric mean: 5 × 10^3^ UFP/cm^3^). In between highest and lowest exposure groups, were catering drivers, cleaning staff and airside security with similar exposure levels (geometric mean: 12–20 × 10^3^ UFP/cm^3^) [87].

Targino et al. (2017): Black carbon (BC) particle concentrations were measured within different micro-environments of 12 airports and on 41 non-smoking commercial flights. Great variability was seen depending on environment measured. 70% of personal exposure during a journey occurred in the airport concourses and during transit to/from the aircraft. 18% was contributed to the waiting time onboard an aircraft with open doors waiting for loading. Largest BC exposure were found during boarding and disembarking (mean BC = 3.78 μg/cm^3^; 25th, 50th, 75th percentiles: 1.29, 2.15, 4.68), at the airport concourse (mean BC = 3.16 μg/cm^3^; 25th, 50th, 75th percentiles: 1.20, 2.15, 4.0) and inside parked aircraft with open doors (mean BC = 2.78 μg/cm^3^; 25th, 50th, 75th percentiles: 0.35, 0.72, 2.33). BC levels were low in the aircraft on the ground with closed doors (mean BC = 0.81 μg/cm^3^; 25th, 50th, 75th percentiles: 0.2, 0.35, 0.72, respectively). Lowest concentration was found during flights in the air [41].

Ren et al. (2018)^a^: The number concentrations and size distributions inside the cabin of an aircraft waiting for take-off were investigated and analyzed in comparison to outdoor UFP and the use of the ground air-conditioning cart (GAC) and environmental control system (ECS), which are used to provide conditioned air between boarding and doors closing to prepare for take-off. The study showed that environmental particle number concentration varied significantly, ranging from 10 to 40 × 10^3^ particles/cm^3^ depending on wind, and take-off and landing activities. When the GAC was on, the indoor particle numbers followed those outdoors, with the ECS providing protection factors for crew and passengers from 1 to 73% for 15–100 nm particles, and from 30 to 47% for 100–600 nm particles. A 40 min wait 100 m downwind of the runway was calculated to be equal to 4 h exposure in a clean urban background environment away from the airport [89].

Ren et al. (2018)^b^: In this study, the potential exposure to passengers as well as indoor airport staff was investigated by measurements in the terminal building of Tianjin Airport in Beijing of CO_2_, PM_2.5_, and UFP concentration and particle size distribution during three seasons. The effects on the indoor air quality of airliner-generated particles penetrating from the outdoor environment through open doors and by heating, ventilation and air-conditioning systems was studied.

PM_2.5_ concentrations in the terminal building varied during the seasons of winter, spring and summer with 337–105-167 μg/m^3^ in the arrival hall, 385–130-170 μg/m^3^ in the departure hall, and 400–156-216 μg/m^3^ in ambient airport air, respectively. These were significant higher levels compared to Chinese standard and WHO annual mean value of 10 μg/m^3^ during all the tested seasons. The indoor environment was significantly affected by the outdoor air levels (Spearman: *p* < 0.01). Particle number concentration in the terminal building displayed two size distribution, with one mode at 30 nm and a mode at 100 nm, which was significantly different from the size distribution measured in a normal urban environment, which had one peak at 100 nm. The study reports particle number concentrations of 1.9–5.9 times higher in the terminal buildings than the concentrations measured in a normal urban environment by different size bins. Measured total UFP exposure during an entire average waiting period (including in the terminal building and airliner cabin) of a passenger was estimated to be equivalent to 11 h of exposure to normal urban emissions [90].

Bendtsen et al. (2019): In this study, the occupational exposure levels to particles was evaluated by measurements at a non-commercial airfield and particles were collected and characterized at a non-commercial airfield and from the apron of a commercial airport.

Electron microscopy showed that the aerosol at the non-commercial airfield appeared to be mainly aggregates of soot, whereas the aerosol at the apron of the commercial airport appeared much more complex dominated by agglomerated soot particles, salt crystals and pollen. At the commercial airport, particles were mainly below 300 nm in diameter and distributed in two modes with geometric mean diameters of < 20 nm and approximately 140 nm. At the non-commercial airfield, two full cycles of a normal workflow of plane leaving, plane arriving and refueling by were recorded in a jet shelter using stationary and portable devices including in the breathing zone of personnel. Average particle number concentration for a full workflow cycle of 170 min were 1.22 × 10^6^ particles/cm^3^. For take-off and landing of one jet plane, average particle number concentrations and mass were 7.7 particles/cm^3^ and 1086 μg/m^3^ and 2.67 particles/cm^3^ and 410 μg/m^3^, respectively. During the main combustion events of plane leaving and arriving, the instruments reached their upper detection limits of 10^6^ particles/cm^3^ (DiSCmini, which measures particle number concentration, mean particle size and lung-deposited surface area) and 10^8^ particles/cm^3^ (ELPI, which monitors real-time particle levels), including in the breathing zone monitor of the personnel. Prevalent particle sizes suggested that the jet engine combustion particles were < 10 nm in aerodynamic diameter [36].

Mokalled et al. (2019): In this study, 48 volatile organic compounds (VOC) from approximately 100 commercial aircraft during real operations of different engine modes at Beirut Rafic Hariri International Airport were assessed to identify specific markers, together with measurements of Jet A-1 kerosene fuel vapors and gasoline exhaust.

Heavy alkanes (C8-C14, mainly n-nonane and n-decane) contributed to 51–64% of the total mass of heavy VOCs emitted by aircraft. Heavy aldehydes (nonanal and decanal) was reported as potential tracers for aircraft emissions due to their exclusive presence in aircraft-related emissions in combination with their absence from gasoline exhaust emissions. Total concentration of heavy alkanes in the ambient air was 47% of the total mass of heavy VOCs measured. No aircraft tracer was identified among the light VOCs (≤ C7). VOC compositions in jet exhaust varied with combustion power, and it was shown that light VOC emissions decrease as the engine power increases. Auxiliary power unit (APU) emissions were identified to be of the same order of magnitude as main engine emissions [93].

Marcias et al. (2019): In this study, occupational exposure to ultrafine particles and noise was investigated for 33 male employees working in an airport taxiway in a smaller Italian airport. Job categories represented were aircraft ground equipment personnel, firefighting officer, flight security agent, and aviation fuel administration staff. Both stationary sampling (ELPI) and personal particle measurements were included. The morphology and chemical composition was determined by EM and EDS, and showed small soot particles in aggregates with sodium, potassium, magnesium, calcium, aluminium, carbon, nitrogen, silicon, oxygen, fluorine, chlorine and sulphur. The maximal UFP number concentration (9.59 × 10^6^ particles/cm^3^) on stationary equipment was measured during support tasks in taxiing and taking off of the aircraft. Median UFP number concentration measured with personal monitors on the 33 operators was 2.44 × 10^3^ particles/cm^3^ and a maximum of 13 × 10^3^ particles/cm^3^. Average size range was 35–103 nm. A significant difference in mean size and distributions was found between job tasks, where flight security officers were exposed to particles with lower mean sizes as compared to aircraft ground equipment operators [92].

### Residential exposure

Westerdahl et al. (2008): Air measurements were carried out in the vicinity of LAX to assess the spread of airport emissions in downwind ambient air to the immediate neighborhood. Ultrafine particle numbers (UFP), size distributions, particle size, black carbon (BC), nitrogen oxides (NOx), and particle-bound PAH were measured. The lowest levels of pollutants were measured upwind of the airport, where UFP ranged from 580 to 3800 particles/cm^3^, black carbon from 0.2 to 0.6 μg/m^3^, and particle-bound PAH from 18 to 36 ng/m^3^. In contrast, at 500 m downwind of the airport, average UFP counts of 50,000 particles/cm^3^ were observed, which were significantly influenced by aircraft operations where peaks were observed. Black carbon, particle-bound PAH, and NO_x_ were also elevated, although not in the same extent, and the authors observed that BC, particle numbers, and NOx levels varied together in similar patterns indicating they were associated with similar sources. Black carbon concentrations varied across the measurement sites, with a mean of 0.3 μg/cm^3^ upwind from the airport, 0.7 μg/cm^3^ downwind from the airport, 1.8 μg/cm^3^ at the taxiway, and 3.8 μg/cm^3^ in the terminal region. Mean PM-PAH levels were 18.2, 24.6, 50.1 and 60.1 ng/m^3^ at the measurement sites, respectively. PM-PAH mean levels measured on two freeways were 47.0 ng/m^3^ and 169.4 ng/m^3^. The maximum UFP measured was 4.8 × 10^6^ particles/m^3^ downwind from a jet aircraft taking off. NOx levels before the take-off were around 8 ppb and increased to 1045 ppb, mostly due to NO. Black carbon rose from approximately 800 to 9550 ng/m^3^, and PM-PAH values increased from 37 to 124 ng/m^3^. Significant variations were observed in particle sizes, where upwind measurements were dominated by particles of 90 nm, and downwind particles were of 10–15 nm in size. The author noted that UFP levels from aircraft were measured to persist up to 900 m from the runways, indicating potential risks for the nearby communities [34].

Lopes et al. (2019): In this study, data is presented from UFP monitoring at several sampling sites in the vicinity of Lisbon Airport in 2017 and 2018, for 19 non-consecutive days. Measurements included sites further away from the airport, under the landing/take-off path. Correlation analysis between air traffic activity and UFP concentrations was conducted and show the occurrence of high UFP concentrations in the airport vicinity. The particle counts increased 18–26 fold at locations near the airport, downwind, and 4-fold at locations up to 1 km from the airport. Results show that particle number increased with the number of flights and decreased with the distance to the airport [91].

Pirhadi et al. (2020): In this study, the contributions of airport activities to particle number concentrations (PNCs) at Amsterdam Schiphol was quantified by use of the positive matrix factorization (PMF) source apportionment model. Various pollutants were measured, including NOx and CO, black carbon, PM2.5 mass, and the number of arrivals and departures were measured for 32 sampling days over 6 months. Airport activities accounted for 79.3% of PNCs divided in aircraft departures, aircraft arrivals, and ground service equipment (GSE) (with contributions of local road traffic, mostly from airport parking areas). Aircraft departures and aircraft arrivals contributed to 46.1 and 26.7% of PNCs, respectively, and were characterized by particle diameters < 20 nm. GSE and local road traffic accounted for 6.5% of the PNCs and were characterized by diameters of around 60–80 nm. Traffic from surrounding freeways was characterized by particles of 30–40 nm and contributed to 18% of PNCs. In comparison, the urban background emissions dominated the mass concentrations with 58.2%, but had the least contribution to PNCs with 2.7% [85].

### Summary of exposure studies

Occupational exposure to increased levels of nanosized particles [36, 85–90, 92], increased levels of PAH including known human carcinogens [52, 83, 84], and black carbon [41] were reported in the literature. Levels of exposure reported in these studies are summarized in Table 1. One study reported that personnel monitors measured higher levels compared to stationary equipment [87], and it was shown that ground support equipment, such as diesel-powered electrical generators and heaters [83] and auxiliary power units [93] contribute significantly to emissions.

Three important main factors were identified which significant influenced occupational exposure: *proximity to emission sources*, where levels were generally higher in close proximity and down-wind to aircraft, *fluctuations in emission levels*, characterized by exposure peak events such as landing- or take-off, and *job type*, where outdoor ground-affiliated work types are at highest risk of exposure. As such, airport personnel can likely be grouped in low (office staff/landside jobs with indoor work, far away from emission sources), medium (catering/cleaning/landside security staff with intermittent outdoor work) and high (baggage handlers/aircraft mechanics/ crew chief) exposure groups.

The majority of studies on the contribution of airport emissions to air pollution in the surrounding environment are physical/chemical studies of particle numbers, mass and related air pollutants, which are reviewed elsewhere as previously described.

More studies reported increased risk of exposure correlating with decreased distance to airports [94–96] and time spent downwind from an airport [97], hence a significant factor for potential health effects for neighboring residential areas based on these studies is *distance to airports,* which relating to wind and atmospheric conditions is an important determinant for pollution levels.

## Health effects

Here we present studies in which direct health effects have been assessed in humans, including in biomonitoring and epidemiological studies, and biological mechanisms-of-action assessed in animal or cell studies. Our main focus is particle exposure, however, studies focusing more on VOC/PAH are also presented.

### Occupational studies

Møller et al. (2017 and 2019): A prospective, occupational cohort study in CPH, encompassing 69,175 men in unskilled positions as baggage handlers or in other outdoor work used register information of socioeconomic, demographic and health data together with a job-exposure matrix was based on GPS measurements within the airport, detailed information on tasks from 1990 to 2012, exposure to air pollution at home, and lifestyle details. Occupational exposure groups were categorized according to work time at the apron, “apron-years” (non-exposed, 0.1–2.9, 3.0–6.9 and ≥ 7 years). The reference group comprised different low-exposure occupational groups [98]. A follow-up study was conducted on an exposed group of 6515 male airport workers at 24–35 years of age in unskilled positions with a reference group of 61,617 men from greater Copenhagen area in unskilled jobs. Exposure was assessed by recordings of time spent on the airport apron and diagnoses of ischemic heart disease and cerebrovascular disease was obtained from the National Patient Register. No associations between cumulative apron-years and the two disease outcomes were found. On the other hand, since the exposed group had a mean age of 24–35 years, a 22-year follow-up may have been too short to detect cardiovascular effects [99].

Lemasters et al. (1997): In this early study, mixed low-level exposure to fuel and solvent was studied in a repeated measures design with male aircraft workers at a military air station serving as their own controls from pre-exposure to 30 weeks post-exposure. The study group consisted of six aircraft sheet metal workers mainly exposed to solvents, adhesives and sealants, six aircraft painters exposed to solvents and paints, 15 jet fueling operations personnel (*n* = 15) responsible for fuel delivery, fueling and defueling aircraft and repairing fuel systems, and 23 workers in the flight line crew exposed to jet fuel, jet exhaust, solvents and paint, and included ground crew and jet engine mechanics. Expired breath analysis was carried out for different trace compounds, but was found to have low values (< 25 parts per billion). An increase in sister chromatid exchange (SCE) compared to pre-exposure was found after 30 weeks of exposure for sheet metal workers (mean SCE per cell increased from 6.5 (SD: 0.8, range: 5.5–7.7) to 7.8 (SD: 0.3, range: 7.4–8.2) and painters (mean SCE per cell increased from 5.9 (SD: 0.7, range: 5.0–6.8) to 6.7 (SD: 1.0, range 5.3–7.8)), indicating exposure to genotoxic substances for these subgroups [100].

Tunnicliffe et al. (1999): In Birmingham International Airport, occupational exposure to aircraft fuel and jet stream exhaust was evaluated in terms of respiratory symptoms and spirometry in 222 full-time employees according to job title. Data was collected by questionnaire and with on-site measurement of lung function, skin prick tests, and exhaled carbon monoxide concentrations. Occupational exposure was assessed by job title, where baggage handlers, airport hands, marshallers, operational engineers, fitters, and engineering technicians were considered as high exposure groups, security staff, fire fighters, and airfield operations managers as medium exposure group, and low exposure groups consisted of terminal and office workers. Upper and lower respiratory tract symptoms were commonly reported in the questionnaire and 51% had one or more positive allergen skin tests. Cough with phlegm and runny nose were found to be significantly associated with high exposure (adj. OR = 3.5, CI: 1.23–9.74; adj. OR = 2.9, CI: 1.32–6.4, respectively). Upper and lower respiratory symptoms were common among exposed workers, but no significant difference was found in lung function. The authors conclude that it is more likely that these symptoms reflect exposure to exhaust rather than fuel [101].

Yang et al. (2003): The aim of this study was to evaluate self-reported adverse chronic respiratory symptoms and acute irritative symptoms among 106 airport workers in risk of exposure to jet fuel or exhaust (jet fuel handlers, baggage handlers, engineers etc.) compared to 305 terminal or office workers (control group) at Kaohsiung International Airport (KIA) in Taiwan. The odds ratio analyses were adjusted for possible confounding factors, such as age, marital status, education, duration of employment, smoking status, and previous occupational exposure to dust or fumes. The prevalence of acute irritative symptoms was not significantly different, whereas chronic respiratory symptoms such as cough (adj. OR = 3.41, CI: 1.26–9.28) and dyspnea (adj. OR = 2.34, CI: 1.05–5.18) were significantly more common among airport workers. The study did not report exposure measurements, but the authors conclude that the expected higher exposure of aviation fuel or exhaust in the ground personnel is the likely explanation for the increased incidence of self-reported chronic respiratory health-effects compared to the office personnel [102].

Whelan et al. (2003): Prevalence of respiratory symptoms among female flight attendants along with teachers was investigated by self-reported questionnaire in comparison to database-derived data on blue collar workers with no known occupational exposures, and it was found that female flight attendants and teachers were significantly more likely to report work related eye (12.4 and 7.4%), nose (15.7 and 8.1%), and throat symptoms (7.5 and 5.7%), and more episodes of wheezing and flu, compared to other female workers (2.9% eye, 2.7% nose, and 1.3% throat symptoms). Female flight attendants were significantly more likely than teachers and controls to report chest illness 3 years in retrospective (flight attendants: 32.9%, teachers: 19.3%, female workers: 7.2%) [103].

Cavallo et al. (2006): In this study, 41 airport employees in jobs with very close proximity to aircraft in service (fitters, airport hands, marshallers, baggage handlers) or in jobs with some proximity to aircraft (security staff, maintenance service personnel, cleaning staff, air field operations managers, runway shuttle drivers) in Leonardo da Vinci airport in Rome were evaluated for exposure to aircraft emissions along with biomarkers of genotoxicity in comparison to a control group of 31 office workers at the same airport. Job tasks in very close proximity to aircraft in service were considered to be high exposure jobs. Urinary PAH metabolites were used as biomarker of endogenous PAH exposure in parallel with PAH analyses of air samples. Exfoliated buccal cells and blood were evaluated for DNA damage, e.g. micronuclei, chromosomal aberrations and sister chromatid exchange (SCE). PAH exposure was measured during 24 h of 5 work days at the airport apron, airport building and terminal/office area from January to February 2005. Total mean of 23 PAHs (particle and vapour) at the apron, airport building and terminal departure area were 27.7, 17.2, and 9.5 μg/m^3^, respectively, with a prevalence of 2–3 ring PAHs with highest levels in the airport apron particularly for 1- and 2-methylnaphthalene and acenaphthene. Urinary PAH metabolite levels were similar for high exposure job groups and controls. The exposed group showed increased SCE (mean number: 4.61 ± 0.80) compared to control group (3.84 ± 0.58) and increased levels of chromosomal aberrations and DNA strand breaks in the Comet assay in both buccal cells and lymphocytes, indicating genotoxic exposures [52].

Radican et al. (2008): A follow-up study of 14,455 workers from 1990 to 2000 evaluated the mortality risk from trichloroethylene and other chemical exposures in aircraft maintenance workers. Relative risk (RR) for exposed compared to unexposed workers were calculated, and positive associations with several cancers were observed, but mortality had not changed substantially since 1990, with increased risk of all-cause mortality (RR = 1.04, CI: 0.98–1.09) or death from all cancers (RR = 1.03, CI: 0.91–1.17) [104].

Erdem et al. (2012): A study group consisting of 43 aircraft fuel maintenance staff, fuel specialists, and mechanics occupationally exposed to JP-8 fuel directly or via engines of jet planes were evaluated for the metabolites 1- and 2-naphthol and creatinine in urine as biomarkers of exposure to jet fuel. In turn, sister chromatid exchange (SCE) and micronuclei were evaluated in blood-derived lymphocytes as biomarkers of genotoxic exposure. Urinary markers and SCE were significantly increased in exposed workers (1-naphthol: 99.01 μmol/mol creatinine; 2-naphthol: 77.29 μmol/mol creatinine), by 10-fold as compared to a control group of 38 employees working in the same area without any work-related exposure to JP-8 fuel [105].

Marie-Desvergne et al. (2016): In this study, exposure to airport nanoparticles and metals was evaluated in airport workers by exhaled breath condensate (EBC) as a non-invasive representative of the respiratory system. EBC was collected from 458 airport workers from Marseille Provence Airport and Roissy Charles de Gaulle Airport in Paris, working directly on the apron (exposed) or in the offices (less exposed). In addition, ambient nanoparticle exposure levels were characterized in terms of particle number concentration, size distribution and by electron microscopy.

The study showed that airport workers were exposed to significantly higher particle numbers (1.0 × 10^4^–2.1 × 10^7^ particles/cm^3^) compared to office staff (10^3^–10^4^ range equivalent to background traffic emissions), although office workers were periodically exposed to peaks of 10^4^–10^5^ when the building doors were open. Airport workers were exposed to significantly smaller particles (mean geometric size: 17.7) compared to office workers (mean geometric size: 23.7). EBC was characterized by volume, total protein content, and a multi-elemental analysis was used to.

measure Na, Al, Cd, and Cr. Particles in EBC were analyzed with dynamic light scattering and electron microscopy (SEM-EDS).

A significantly higher concentration of Cd was found in apron worker EBC (mean: 0.174 ± 0.326 μg/l) in comparison with office workers (mean: 0.108 ± 0.106 μg/l). Particulate content in EBC was confirmed by DLS and SEM-EDS, but no differences were found between the two study groups, and measured EBC particle contents did not correlate with ambient exposure levels [88].

### Studies on effects of residential exposure to airport emissions

Visser et al. (2005): In this population-based study, it was investigated if the residents living around Amsterdam Schiphol Airport were at higher risk of developing cancer compared to the general Dutch population. The regional cancer registry was used, estimating the cancer incidence from 1988 to 2003 in the population residing near the airport compared to the national cancer incidence. The exposure was defined by aircraft noise and postal code areas, as historical data on ambient air pollution were unavailable. The study did not include information on lifestyle factors, and therefore, did not control for smoking and other potential confounders. A core zone closest to the airport and a remaining ring zone was studied. Thirteen thousand two hundred seven cancer cases were identified in the study area, and a significant increase in the incidence of hematological cancers (standardized incidence ratio, SIR = 1.12, CI: 1.05–1.19) was found, mainly due to non-Hodgkin lymphoma (SIR = 1.22, 95% CI: 1.12, 1.33) and acute lymphoblastic leukemia (SIR = 1.34, CI: 0.95, 1.83). Respiratory system cancer incidence was significantly decreased (SIR = 0.94, CI: 0.90, 0.99), due to the low rate in males (SIR = 0.89). The study concludes that the overall cancer incidence in the residential areas closest to Amsterdam Schiphol Airport was similar to the national incidence. The increase in the risk of hematological cancers could not be explained by higher levels of ambient air pollution in the area [106].

Lin et al. (2008): In this cross-sectional study, it was assessed whether residents living near commercial airports had increased rates of hospital admissions due to respiratory diseases compared to those living further away. The study included all residents living within 12 miles from the center of each of three airports (Rochester in Rochester, LaGuardia in New York City and MacArthur in Long Island). Hospital admission data were collected by the New York State Department of Health for all residents who were hospitalized for asthma, chronic bronchitis, emphysema, chronic obstructive pulmonary disease and, for children aged 0–4 years, bronchitis and bronchiolitis during 1995–2000. Exposure indicators were distance from the airport and dominant wind patterns from the airports.

The relative risks of hospital admissions due to respiratory conditions for residents living < 5 miles from the airport were 1.47 (CI: 1.41–1.52) for Rochester and 1.38 (CI: 1.37–1.39) for LaGuardia, as compared to those living > 5 miles from the airports. No differences were observed for MacArthur airport. When considering hospital admission rates by distance for 12–1 miles towards the airports, a significant trend of increasing hospital admissions with closer distance to the airport was observed for the Rochester airport. The authors reported a stronger effect for traditionally lower socio-economic groups [94], which may be of more relevance in the US, due to the medical insurance system.

Habre et al. (2018): In this study, 22 non-smoking volunteers with mild to moderate asthma were recruited to do scripted mild walking activity in parks inside or outside a zone of high airport-related ultrafine particle exposure downwind of LAX. Physiological parameters were measured before and after exposure, and the study was conducted as a cross-over study, such that the participants served as their own controls. Personal exposure to black carbon, PAH, ozone, and PM_2.5_ were measured and combined with source appointment analysis and health models. A difference in PM exposure was found between the high (mean particle number concentration of 53,342 particles/cm^3^ and mean particle size of 28.7 nm) and the low exposure zone (mean particle number concentration of 19,557 particles/cm^3^ and mean particle size of 33.2 nm). It was reported that IL-6 levels in blood were increased after the walk in the high exposure zone compared to the low exposure zone. Airport-related PM was distinguished from roadway traffic emissions by principal component analysis, and increase of airport-related PM was significantly associated with increased IL-6 levels [107].

Amsterdam Schiphol report (2019): Based on three studies with 191 primary school children from residential areas near Schiphol Airport, 21 healthy adults living adjacent to the airport [108], and an in vitro study [109], respectively, this Dutch report (not subjected to peer review) describes the findings of reduced lung function in children and adults following higher short-term exposure to ultrafine particles near Schiphol Airport. On days with high exposure, children suffered more from respiratory complaints and used more medicine. In the adults, short-term reductions in heart function were also found. The authors note that these effects may be larger for individuals already suffering from medical conditions. The authors point out that the effects are results of ultrafine particles from both air and road traffic, and that there are no indications that health effects of air traffic emissions are different from those caused by road traffic [59].

Lammers et al. 2020: This study investigated the health effects of controlled short-term exposure of 21 healthy non-smoking volunteers aged 18–35 years to UFP near Shiphol Airport Amsterdam. The volunteers were exposed 2–5 times to ambient are for 5 h while cycling. Cardiopulmonary outcomes such as spirometry, forced exhaled nitric oxide, electrocardiography and blood pressure were measured before and after exposure, and compared to measured total- and size-specific particle number concentrations (PNC). Average PNC was 53,500 particles/cm^3^ (range 10,500–173,200). Increase in exposure to UFP was associated with a decrease in FVC and a prolongation of the corrected QT interval, which were associated with particle sizes < 20 nm (UFP from aviation), but not with particles > 50 nm (UFP from road traffic). Although the effects were relatively small and measured after single exposures of 5 h in young healthy adults [108], such effects could be important in susceptible sub-populations.

### Animal studies and in vitro studies

Ferry et al. (2011): Immature primary human monocyte-derived dendritic cells (DCs) from healthy donor blood were exposed for 18 h to different doses of experimental jet exhaust particles in absence or presence of *E. coli* lipopolysaccharides (LPS). Antigen-presenting and stimulatory molecules were measured along with tumor necrosis factor (TNFα) and IL-10. The effects were assessed on immature and mature DCs as well as on cells during the maturation process.

The primary particles collected from the jet exhaust by direct impaction were found to be spherical and carbonaceous primary particles of ~ 10 nm and aggregates up to ~ 93 nm. No toxic effects were observed for doses below of 100 μg/mL jet engine particles. Maturation of immature dendritic cells by LPS stimulation induced a significant 500-fold increase in TNFα and 30-fold increase in IL-10. Immature dendritic cells produced low amounts of TNFα (fold change from LPS: 0.006) and IL-10 (fold change from LPS: 0.11), which increased non-significantly upon stimulation with particles (fold change from LPS: TNFα: 0.11, IL-10: 0.19). However, simultaneous exposure to LPS and a high particle dose of 100 μg/ml induced a 2-fold increase in TNFα production compared to LPS-maturation (*p* = 3 × 10^− 5^). Different activation patterns were seen for the expression of HLA DR and CD86, which are dendritic cell maturation markers. It was concluded that jet exhaust particles may act as adjuvants to endotoxin-induced dendritic cell maturation, which may influence potential effects on human health [110].

Shirmohammadi et al. (2018): PM_0.25_ collected at the vicinity of Los Angeles Airport (LAX) and from central Los Angeles (LA) close to and downwind from major freeways, from stationary sampling stations used for air quality control, were investigated. The particles were subjected to source allocation analyses of elements and carbon contents (see Introduction), and ROS formation was compared in rat alveolar macrophage cells (NR8383).

ROS activity measured as units of Zymosan equivalents were normalized by total PM0.25 mass to represent the intrinsic toxicity of the particles, and this mass-normalized ROS activity was similar for LAX (4600.93 ± 1516.98 μg Zymosan/mg PM) and central LA (4391.22 ± 1902.54 μg Zymosan/mg PM). According to the authors, volume-normalization of the ROS activity can be used as a metric for comparison of inhalation exposures, as an indicator of exposure severity. A slightly higher PM0.25 mass concentration in central LA meant overall similar volume-normalized ROS activity levels with no significant difference between the observed averages (LAX: 24.75 ± 14.01 μg Zymosan/m^3^, central LA: 27.77 ± 20.32 μg Zymosan/m^3^). Thus, there were similar levels of ROS activity and similar toxic potential of the PM in the vicinity of LAX and in the vicinity of freeways in central LA [49].

He et al. (2018): PM_0.25_ collected at Los Angeles Airport (LAX) and from central Los Angeles (LA) close to and downwind from major freeways (similar collection sites as in [49]) were investigated and compared. Particles were source-allocated by analyzing elements (see Introduction). Particles collected at LAX were primarily associated with aircraft emissions, and particles from central LA with urban traffic, road and dust emissions. The reactive oxygen species (ROS) potential was evaluated intracellularly in human bronchial epithelial cells (16HBE) after 1, 2, and 4 h of exposure, and IL-6, IL-8 and TNF were measured as markers of inflammation.

Exposure of 16HBE cells to 10 μg/mL particles produced significantly elevated ROS levels for both samples compared to unexposed cells. Particles from central LA generated slightly more ROS than LAX samples per mass unit, and both were at negative control level after 20 h recovery. ROS potential in PM from both airport and central LA correlated with some of the measured traffic-related transition metals (Fe and Cu). Particles from LAX induced increased expression of IL-6, IL-8 and TNFα compared to the negative control (1.7, 1.8, and 1.4-fold, respectively), whereas central LA-particles induced slightly lower expressions (1.3, 1.3, and 1.1-fold, respectively). Hence, overall LAX particles had similar inflammatory potency as particles from central LA, showing that airport PM_0.25_ contributions to urban emission PM pollution possess similar inflammatory properties [50].

Jonsdottir et al. (2019): In this study, aerosol was collected from the world’s most used aircraft turbine (CFM56–7B26, run-in and airworthy) in a test cell at Zurich Airport. The test cell is open to the ambient environment and the aerosol was collected from both standard Jet A-1 fuel and a HEFA fuel blend. The toxicity of the non-volatile PM emissions was studied by direct particle deposition onto air-liquid interface cultures of human bronchial epithelial cells (BEAS-2B).

Cytotoxicity was evaluated by the release of cytosolic LDH from damaged cells, expression of the oxidative stress marker HMOX-1 and inflammatory cytokines IL-6 and IL-8.

Single, short-term (1 h) exposure to PM increased cell membrane damage, lead to oxidative stress and increased pro-inflammatory cytokines in bronchial epithelial cells, depending on fuel type and combustion conditions from which the particles were produced. PM from conventional fuel at ground-idle conditions was most potent, and the authors comment that PM from aircraft turbine exhaust may be a risk to respiratory health, also by making airway epithelia vulnerable to secondary exposure of other air pollution compounds and pathogens [111].

Bendtsen et al. (2019): In this study, the toxicity of particles collected in a commercial and a non-commercial airport were evaluated in vivo by intratracheal instillation in mice (see section 2.3 for occupational exposure measurements). Adult female C57BL/6 mice were exposed to 6, 18, and 54 μg particles/mouse dispersed in Nanopure water by sonication. The exposure doses were calculated on the basis of worst case scenario: of the maximum exposure level measured at the non-commercial airport of 1086 μg/m^3^ at the peak event of plane departure, 9.6% were estimated to deposit in the alveolar lung regions. This was adjusted to the volume of a mouse lung and to 8 h of work, estimating exposure of 4, 12, and 39 days of work, respectively. Control mice were exposed to Nanopure water, and positive controls were carbon black Printex90 nanoparticles and SRM2975 diesel particles. Exposed mice were euthanized on day 1, 28, and 90 post-exposure. Inflammation was measured as inflammatory cell influx in bronchoalveolar lavage fluid as well as by the acute-phase response marker *serum amyloid A* (*Saa*) in lung (mRNA), liver (mRNA) and blood (protein). Genotoxicity was assessed by the comet assay on lung and liver tissue and cells from the bronchoalveolar lavage fluid. Analysis of the particles by scanning and transmission electron microscopy showed small primary particles and agglomerates of soot, which appeared uniform for non-commercial airport particles (mainly from jet engine emissions) and more heterogenous for the commercial airport particles (emissions from aircraft, ocean, traffic and background). Pulmonary exposure to particles from both airports induced genotoxicity and dose-dependent acute phase response, and inflammation at same levels as standard diesel exhaust particles and carbon black nanoparticles [36].

He et al. 2020: In this study, UFPs from aviation or road traffic emissions were collected near the major international airport, Amsterdam-Schiphol airport (AMS), along with UFPs from an aircraft turbine engine at low and full thrust. The toxicity of the particles was tested in human bronchial epithelial cells (Calu-3) combined with an air-liquid interface (ALI) system with exposure to UPFs at low doses from 0.09 to 2.07 μg/cm^2^. Cell viability, cytotoxicity and IL-6 and -8 secretion were assessed after 24 h exposure. Cell viability was < 80% for all doses. LDH release as measure of cytotoxicity was observed at the highest exposure dose around 1.5 μg/cm^2^ together with increased production of IL-6 and IL-8 compared to control exposure (blank filter extraction or re-suspension solution). It was concluded that airport and road traffic UFP as well as UFP samples from the turbine engine had similar inflammatory properties [109].

### Summary of health effect studies

Increased levels of metabolites in urine as biomarkers of internal exposure to jet fuel [105] were reported in biomonitoring studies of occupational exposure to airport emissions. Exposure to airport emissions was associated with increased levels of biomarkers of genotoxicity, in terms of increased levels of SCE [52, 100, 105] and DNA strand breaks in the Comet assay [52], which indicates exposure to genotoxic and potential carcinogenic agents in the emissions. In turn, there were occupational studies reporting increased levels of self-reported respiratory complaints [101–103].

We identified a limited number of studies and one report reporting correlations between airport emission levels and health effects of residents in the vicinity of airports: Aircraft emission levels were associated with increased hospitalization for asthma, respiratory, and heart conditions especially in susceptible subgroups such as children below 5 years of age, elderly above 65 years of age [66, 94] and lower socioeconomic groups [97, 112]. A Dutch report on Schiphol similarly reported that school children and adults took more medication and had more respiratory complaint on days with increased exposure to aircraft emissions and concludes that health effects of air traffic emissions are similar to those caused by road traffic [59]. A biomonitoring study showed increased blood levels of the inflammatory marker IL-6 in volunteers with mild to moderate asthma after a walk in a zone with high levels of aircraft emissions [107]. It is well-known that other types of air pollution including diesel exhaust cause morbidity and mortality [113]. Taken together, these results suggest that the exposure to aircraft emissions induce pulmonary and systemic inflammation, which potentially contributes to cancer, asthma, respiratory and coronary heart disease.

Five mechanistic studies on the toxicity of airport particles were identified, one animal study in mice and four cell studies: Airport particles were reported to act as adjuvants in the activation of inflammatory cells or pathways [110] and induce pro-inflammatory cytokines [111]. Airport particles were shown to have similar inflammatory potency and similar ability to induce DNA damage as traffic emission particles [50], such as diesel exhaust particles [36]. In turn, airport particles induced significant levels of the biomarker Saa following intratracheal instillation in mice, associated with risk of cardiovascular disease [36], and they have the potential to generate ROS at similar levels as traffic emission particles [49, 50]. Thus, the conclusions from these in vitro and in vivo studies support the overall concern addressed in previous sections that airport emission particles are capable of inducing toxic responses comparable to the responses observed for other air pollution particles such as diesel exhaust particles.

## Discussion

Although a range of kerosene-based aircraft fuel types are in use, they are overall similar in chemical composition [24, 29]. Kerosene lies between the distillated crude oil fractions of gasoline (gasoline combustion exhaust, IARC group 2b) and diesel (diesel combustion exhaust, IARC group 1) and the carcinogenic potential of jet fuel combustion products could be anticipated given the reported similarities to diesel exhaust particles. We highlight two important reported characteristics of airport particles:

- The majority of non-volatile airport emission particles are carbonaceous and aircraft engines emit large amounts of nanoparticles, which are dominated by very small particles of < 20 nm, which form aggregates/agglomerates in ambient air
- Particle numbers near airports are significantly higher than away from airports and jet engines are a significant source of UFP in ambient air. The highest concentrations of UFP are measured downwind of aircraft

The reported PAH levels [52, 83, 84] were all below the current Danish occupational exposure limit of 200 μg/m^3^. One study reported BC levels at the apron of 3.78 μg/m^3^ and particle levels was overall reported to be between ~ 10^3^ and 10^8^ particles/cm^3^ for exposed airport personnel (Table 1). The new exposure limit for diesel exhaust particles in EU is defined by the elemental carbon (EC) level and is 50 μg EC/m^3^ [114]. The Netherland recently endorsed an OEL for diesel exhaust particles at 0.01 mg/m^3^ measured as respirable EC. This was based on socioeconomic considerations and the Dutch prohibition risk level (OEL) is at 1.03 μg EC/m^3^ [115], a level corresponding to 4 extra death cases of lung cancer per 1000 exposed, for 40 years of occupational exposure. Thus, the reported BC level [41] are well below the new EU OEL for diesel exhaust as well as the Dutch OEL, but exceed the Dutch prohibition risk level. Recently published data on the dose-response relationship between exposure to diesel exhaust particles and lung cancer in epidemiological studies estimated that occupational exposure to 1 μg/m^3^ EC would cause 4 to17 excess lung cancer cases per 10,000 exposed [80, 81].

The particle exposure levels can be compared to nanoparticle reference values used in The Netherlands, Germany and Finland as a provisional substitute when nano-specific OELs or DNELs for engineered nanoparticles are not available [116]. For low density insoluble nanomaterials such as carbon-based nanoparticles, the reference value is 40,000 particles/cm^3^. Compared to this reference value for engineered nanoparticles, the reported occupational exposure levels are high for some job groups.

Significant variations in emission levels are observed between airports, depending on factors such as size, type, location, and wind direction. However, the closer to the source of emissions, the higher the exposure. Proximity to exposure peak events such as landing and take-off is also an important determinant of high exposure. This is evident from the combined literature of occupational exposure measurements and ambient air measurements in residential areas around airports. As such, the highest levels of occupational exposure is found for airport personnel working at the apron, in close proximity to running jet engines. Airport personnel can likely be grouped in low (office staff/landside jobs with indoor work, far away from emission sources), medium (catering/cleaning/landside security staff with intermittent outdoor work) and high (baggage handlers/aircraft mechanics, crew chiefs) exposure groups [52, 86–88, 92, 98, 100–102]. To reduce occupational exposure, emission sources can be moved, the distance to emission sources can be increased, time spent in proximity to emission sources can be reduced and personal protection equipment can be used during peak exposures. Personal exposure may be higher than measured by stationary monitors, and thus, routine monitoring of personal exposure levels could be suggested.

Workplace experts, airport leaders and personnel groups have the necessary intrinsic knowledge and experience to suggest feasible, realistic options for reducing the exposure for specific job functions at individual airports.

The similarity of airport emission particles with diesel exhaust particles and pure carbon nanoparticles, with respect to physico-chemical properties as well as specific toxicological parameters was demonstrated in the animal study from our laboratory [36], and a growing number of studies report similar toxicity and health effects of emissions from airports and traffic. Airport emission particles likely have similar physico-chemical properties as diesel exhaust particles even though the primary particle size of jet engine emissions is somewhat smaller than the primary size of diesel exhaust particles. Diesel exhaust is classified as carcinogenic to humans by IARC [69], cause lung cancer, systemic inflammation, and inflammatory responses in the airways [70].

Aircraft emissions are associated with biomarkers of exposure, biomarkers of disease and health outcomes both for exposed workers [36, 41, 52, 83, 84, 86–90, 92, 100–103, 105] and for the general population living down-wind of airports [59, 66, 94–97, 107, 112]. Occupational exposure to aircraft emissions were associated with:

- Biomarkers of exposure to jet fuel emissions
- Biomarkers of genotoxic exposure
- Self-reported respiratory distress

The reported adverse effects correlate with effects demonstrated in animal studies and in in vitro studies, where aircraft emission particles caused inflammation [50, 110, 111], acute phase response [36], reactive oxygen species [49, 50] and DNA damage [36], which are biomarkers of risk of cancer, cardiovascular disease and respiratory disease. This supports the notion of a causal relationship between exposure to airport emissions and the observed health effects. Although mechanistic studies on airport emissions are scarce, knowledge from other closely related scientific areas still applies, such as particle toxicity, carcinogenicity/toxicity of VOCs and OPEs and epidemiological studies of health effects caused by air pollution [117].

Another relevant concern to raise in this context is the adverse health effects of low-level chronic occupational exposure to these chemicals, which is difficult to study [118]. OPEs have been associated with adverse health effects reported from cabin crew and pilots after occupational exposure to bleed air and fume events during flights, with symptoms of respiratory illness and neurological effects [119]. The dominant OPE used in lubrication oil is tri-cresyl phosphate (TCP), which are among the highly neurotoxic OPEs [120]. It has been suggested that brain exposure may occur via inhalation of circulating small jet particles associated with OPEs, crossing the blood-brain barrier [121] – neurotoxic effects of OPEs may also be an understudied occupational risk of apron staff.

It has been shown that air pollutants worsen pre-existing diseases, such as allergy or other inflammatory (airway) or cardiovascular conditions [2–4, 122–124]. One example is a study examining the relationship between personal exposure to traffic emissions and acute respiratory health in school children with asthma residing in the Bronx, New York, which have the highest asthma incidence in New York City and state [125]. Personal samples of PM_2.5_, including the EC fraction, were collected 24 h daily for 40 school children with asthma from four schools, with spirometry and symptoms assessed several times daily. The study found increased relative risks of different airway symptoms, such as wheeze (RR = 1.45, CI: 1.03–2.04), shortness of breath (RR = 1.41, CI: 1.01–1.99), with relative risk of total symptoms of 1.30 (CI: 1.04–1.62). Interestingly, the symptoms were associated with increase in average 2-day school site and personal EC levels, but not mass of PM_2.5_ [125]. As such, as demonstrated in asthmatic volunteers, residents living near airports, and supported by inflammatory effects shown in available in vitro studies, airport UFP and associated pollutants are, in addition to their direct adverse effects, likely to have the ability of worsen pre-existing disease.

## Conclusion

The reported adverse health effects of jet engine emissions are similar to those caused by exposure to diesel exhaust and air pollution. However, given the lack of consensus on optimal measurement methods, equipment and quality control for near- and far field airport emissions and human risk assessments markers, more studies of exposure and of toxicological mechanisms are necessary.

These drawbacks are summarized efficiently by Lighty et al. in their paper on combustion compounds and health: “*There is a need for better integration of the combustion, air pollution control, atmospheric chemistry, and inhalation health research communities. Epidemiology has demonstrated that susceptible individuals are being harmed by ambient PM. Particle surface area, number of ultrafine particles, bioavailable transition metals, polycyclic aromatic hydrocarbons (PAH), and other particle-bound organic compounds are suspected to be more important than particle mass in determining the effects of air pollution. Time- and size-resolved PM measurements are needed for testing mechanistic toxicological hypotheses, for characterizing the relationship between combustion operating conditions and transient emissions, and for source apportionment studies to develop air quality plans*” [24].

Based on the accumulated knowledge so far, measures to reduce occupational exposure and emission levels at airports should be increased.

## Supplementary Information

**Additional file 1.**

## Data Availability

Data sharing not applicable to this article as no datasets were generated or analyzed during the current study.

## References

[1] Utell MJ, Frampton MW (2000). Acute health effects of ambient air pollution: The ultrafine particle hypothesis. J Aerosol Med-Depos Clear Eff Lung.

[2] Pope CA, Turner MC, Burnett RT, Jerrett M, Gapstur SM, Diver WR, Krewski D, Brook RD (2015). Relationships Between Fine Particulate Air Pollution, Cardiometabolic Disorders, and Cardiovascular Mortality. Circ Res.

[3] Kunzli N, Bridevaux PO, Liu LJS, Garcia-Esteban R, Schindler C, Gerbase MW, Sunyer J, Keidel D, Rochat T, Team S (2009). Traffic-related air pollution correlates with adult-onset asthma among never-smokers. Thorax.

[4] Neupane B, Jerrett M, Burnett RT, Marrie T, Arain A, Loeb M (2010). Long-Term Exposure to Ambient Air Pollution and Risk of Hospitalization with Community-acquired Pneumonia in Older Adults. Am J Res Crit Care Med.

[5] Masiol M, Harrison RM (2014). Aircraft engine exhaust emissions and other airport-related contributions to ambient air pollution: A review. Atmos Environ.

[6] Harrison RM, Masiol M, Vardoulakis S (2015). Civil aviation, air pollution and human health. Environ Res Lett.

[7] Hsu H-H, Adamkiewicz G, Houseman EA, Zarubiak D, Spengler JD, Levy JI (2013). Contributions of aircraft arrivals and departures to ultrafine particle counts near Los Angeles International Airport. Sci Total Environ.

[8] Winther M, Kousgaard U, Ellermann T, Massling A, Nøjgaard JK, Ketzel M (2015). Emissions of NOx, particle mass and particle numbers from aircraft main engines, APU's and handling equipment at Copenhagen Airport. Atmos Environ.

[9] Stacey B (2019). Measurement of ultrafine particles at airports: A review. Atmos Environ.

[10] Ritchie G, Still K, Rossi J, Bekkedal M, Bobb A, Arfsten D (2003). Biological and health effects of exposure to kerosene-based jet fuels and performance additives. Journal of toxicology and environmental health Part B. Crit Rev.

[11] Mattie DR, Sterner TR (2011). Past, present and emerging toxicity issues for jet fuel. Toxicol Appl Pharmacol.

[12] Pleil JD, Smith LB, Zelnick SD (2000). Personal exposure to JP-8 jet fuel vapors and exhaust at air force bases. Environ Health Perspect.

[13] Egeghy PP, Hauf-Cabalo L, Gibson R, Rappaport SM (2003). Benzene and naphthalene in air and breath as indicators of exposure to jet fuel. Occup Environ Med.

[14] Wang S, Young RS, Sun NN, Witten ML (2002). In vitro cytokine release from rat type II pneumocytes and alveolar macrophages following exposure to JP-8 jet fuel in co-culture. Toxicology.

[15] Pfaff J, Parton K, Clark Lantz R, Chen H, Hays AM, Witten ML (1995). Inhalation exposure to jp-8 jet fuel alters pulmonary function and substance p levels in fischer 344 rats. J Appl Toxicol.

[16] Pfaff JK, Tollinger BJ, Lantz RC, Chen H, Hays AM, Witten ML (1996). Neutral endopeptidase (NEP) and its role in pathological pulmonary change with inhalation exposure to JP-8 jet fuel. Toxicol Ind Health.

[17] Fechter LD, Gearhart C, Fulton S, Campbell J, Fisher J, Na K, Cocker D, Nelson-Miller A, Moon P, Pouyatos B (2007). JP-8 jet fuel can promote auditory impairment resulting from subsequent noise exposure in rats. Toxicol Sci.

[18] Fechter LD, Fisher JW, Chapman GD, Mokashi VP, Ortiz PA, Reboulet JE, Stubbs JE, Lear AM, McInturf SM, Prues SL (2012). Subchronic JP-8 jet fuel exposure enhances vulnerability to noise-induced hearing loss in rats. J Toxicol Environ Health A.

[19] Kaufman LR, LeMasters GK, Olsen DM, Succop P (2005). Effects of concurrent noise and jet fuel exposure on hearing loss. J Occup Environ Med.

[20] Fife TD, Robb MJA, Steenerson KK, Saha KC (2018). Bilateral Vestibular Dysfunction Associated With Chronic Exposure to Military Jet Propellant Type-Eight Jet. Fuel.

[21] Harris DT, Sakiestewa D, Titone D, Robledo RF, Young RS, Witten M (2000). Jet fuel-induced immunotoxicity. Toxicol Ind Health.

[22] Harris DT, Sakiestewa D, Titone D, Young RS, Witten M (2002). JP-8 jet fuel exposure results in immediate immunotoxicity, which is cumulative over time. Toxicol Ind Health.

[23] Mattie DR, Sterner TR, Reddy G, Steup DR, Zeiger E, Wagner DJ, Kurtz K, Daughtrey WC, Wong BA, Dodd DE (2018). Toxicity and occupational exposure assessment for Fischer-Tropsch synthetic paraffinic kerosene. J Toxicol Environ Health A.

[24] Lighty JS, Veranth JM, Sarofim AF (2000). Combustion Aerosols: Factors Governing Their Size and Composition and Implications to Human Health. J Air Waste Manag Assoc.

[25] Hammes K, Schmidt MWI, Smernik RJ, Currie LA, Ball WP, Nguyen TH, Louchouarn P, Houel S, Gustafsson Ö, Elmquist M, et al. Comparison of quantification methods to measure fire-derived (black/elemental) carbon in soils and sediments using reference materials from soil, water, sediment and the atmosphere. Glob Biogeochem Cycles. 2007;21(3):GB3016. 10.1029/2006GB002914.

[26] Singh A, Rajput P, Sharma D, Sarin MM, Singh D (2014). Black Carbon and Elemental Carbon from Postharvest Agricultural-Waste Burning Emissions in the Indo-Gangetic Plain. J Adv Meteorol.

[27] Costabile F, Angelini F, Barnaba F, Gobbi GP (2015). Partitioning of Black Carbon between ultrafine and fine particle modes in an urban airport vs. urban background environment. Atmos Environ.

[28] Keuken MP, Moerman M, Zandveld P, Henzing JS, Hoek G (2015). Total and size-resolved particle number and black carbon concentrations in urban areas near Schiphol airport (the Netherlands). Atmos Environ.

[29] Mazaheri M, Johnson GR, Morawska L (2011). An inventory of particle and gaseous emissions from large aircraft thrust engine operations at an airport. Atmos Environ.

[30] Stacey B, Harrison RM, Pope F (2019). Evaluation of ultrafine particle concentrations and size distributions at London Heathrow Airport. Atmos Environ.

[31] Liati A, Schreiber D, Alpert PA, Liao Y, Brem BT, Corral Arroyo P, Hu J, Jonsdottir HR, Ammann M, Dimopoulos Eggenschwiler P (2019). Aircraft soot from conventional fuels and biofuels during ground idle and climb-out conditions: Electron microscopy and X-ray micro-spectroscopy. Environ Pollut.

[32] Shirmohammadi F, Sowlat MH, Hasheminassab S, Saffari A, Ban-Weiss G, Sioutas C (2017). Emission rates of particle number, mass and black carbon by the Los Angeles International Airport (LAX) and its impact on air quality in Los Angeles. Atmos Environ.

[33] Campagna M, Frattolillo A, Pili S, Marcias G, Angius N, Mastino CC, Cocco P, Buonanno G (2016). Environmental exposure to ultrafine particles inside and nearby a military airport. Atmosphere.

[34] Westerdahl D, Fruin SA, Fine PL, Sioutas C (2008). The Los Angeles International Airport as a source of ultrafine particles and other pollutants to nearby communities. Atmos Environ.

[35] Canepari S, Padella F, Astolfi ML, Marconi E, Perrino C (2013). Elemental Concentration in Atmospheric Particulate Matter: Estimation of Nanoparticle Contribution. Aerosol Air Qual Res.

[36] Bendtsen KM, Brostrøm A, Koivisto AJ, Koponen I, Berthing T, Bertram N, Kling KI, Dal Maso M, Kangasniemi O, Poikkimäki M (2019). Airport emission particles: exposure characterization and toxicity following intratracheal instillation in mice. Particle Fibre Toxicol.

[37] Rahim MF, Pal D, Ariya PA (2019). Physicochemical studies of aerosols at Montreal Trudeau Airport: The importance of airborne nanoparticles containing metal contaminants. Environ Pollut.

[38] Vander Wal RL, Bryg VM, Huang C-H (2014). Aircraft engine particulate matter: Macro- micro- and nanostructure by HRTEM and chemistry by XPS. Combustion Flame.

[39] Moore RH, Thornhill KL, Weinzierl B, Sauer D, D’Ascoli E, Kim J, Lichtenstern M, Scheibe M, Beaton B, Beyersdorf AJ (2017). Biofuel blending reduces particle emissions from aircraft engines at cruise conditions. Nature.

[40] Agrawal H, Sawant AA, Jansen K, Wayne Miller J, Cocker DR (2008). Characterization of chemical and particulate emissions from aircraft engines. Atmos Environ.

[41] Targino AC, Machado BLF, Krecl P (2017). Concentrations and personal exposure to black carbon particles at airports and on commercial flights. Transport Res.

[42] Fushimi A, Saitoh K, Fujitani Y, Takegawa N (2019). Identification of jet lubrication oil as a major component of aircraft exhaust nanoparticles. Atmos Chem Phys.

[43] Li W, Wang Y, Kannan K (2019). Occurrence, distribution and human exposure to 20 organophosphate esters in air, soil, pine needles, river water, and dust samples collected around an airport in New York state, United States. Environ Int.

[44] Solbu K, Daae HL, Thorud S, Ellingsen DG, Lundanes E, Molander P (2010). Exposure to airborne organophosphates originating from hydraulic and turbine oils among aviation technicians and loaders. J Environ Monitor.

[45] Harrison V, Mackenzie Ross SJ (2016). An emerging concern: Toxic fumes in airplane cabins. Cortex.

[46] Michaelis SBJ, Howard CV (2017). Aerotoxic syndrome: a new occupational disease?. Public Health Panorama.

[47] Boyle KA (1996). Evaluating particulate emissions from jet engines: analysis of chemical and physical characteristics and potential impacts on coastal environments and human health. Transport Res Record.

[48] Abegglen M, Brem BT, Ellenrieder M, Durdina L, Rindlisbacher T, Wang J, Lohmann U, Sierau B (2016). Chemical characterization of freshly emitted particulate matter from aircraft exhaust using single particle mass spectrometry. Atmos Environ.

[49] Shirmohammadi F, Lovett C, Sowlat MH, Mousavi A, Verma V, Shafer MM, Schauer JJ, Sioutas C (2018). Chemical composition and redox activity of PM0.25 near Los Angeles International Airport and comparisons to an urban traffic site. Sci Total Environ.

[50] He R-W, Shirmohammadi F, Gerlofs-Nijland ME, Sioutas C, Cassee FR (2018). Pro-inflammatory responses to PM0.25 from airport and urban traffic emissions. Sci Total Environ.

[51] Turgut ET, Gaga EO, Jovanovic G, Odabasi M, Artun G, Ari A, Urosevic MA (2019). Elemental characterization of general aviation aircraft emissions using moss bags. Environ Sci Pollut Res Int.

[52] Cavallo D, Ursini CL, Carelli G, Iavicoli I, Ciervo A, Perniconi B, Rondinone B, Gismondi M, Iavicoli S (2006). Occupational exposure in airport personnel: Characterization and evaluation of genotoxic and oxidative effects. Toxicology.

[53] Lai C-H, Chuang K-Y, Chang J-W (2013). Characteristics of nano−/ultrafine particle-bound PAHs in ambient air at an international airport. Environ Sci Pollut Res.

[54] Chen Y-C, Lee W-J, Uang S-N, Lee S-H, Tsai P-J (2006). Characteristics of polycyclic aromatic hydrocarbon (PAH) emissions from a UH-1H helicopter engine and its impact on the ambient environment. Atmos Environ.

[55] European Commission (2001). Ambient air pollution by Polycyclic Aromatic Hydrocarbons (PAH). Position Paper.

[56] Zanoni I, Ostuni R, Marek LR, Barresi S, Barbalat R, Barton GM, Granucci F, Kagan JC (2011). CD14 Controls the LPS-Induced Endocytosis of Toll-like Receptor 4. Cell.

[57] Federal Aviation Administration (2003). Select Resource Materials and Annotated Bibliography on the Topic of Hazardous Air Pollutants (HAPs) Associated with Aircraft, Airports and Aviation. Federal Aviation Administration Office of Environment and Energy.

[58] Mokalled T, Gérard JA, Abboud M, Liaud C, Nasreddine R, Le Calvé S (2019). An assessment of indoor air quality in the maintenance room at Beirut-Rafic Hariri International Airport. Atmos Pollut Res.

[59] Janssen N, Lammer M, Maitland-van de Zee A, van de Zee S, Keuken R, Blom M, van den Bulk P, van Dinther D, Hoek G, Kamstra K, et al. Onderzoek naar de gezondheidseffecten van kortdurende blootstelling aan ultrafijn stof rond Schiphol 2019–0084 edn. The Nederlands: RIVM official reports; 2019. p. 188.

[60] Stafoggia M, Cattani G, Forastiere F, Di Menno di Bucchianico A, Gaeta A, Ancona C (2016). Particle number concentrations near the Rome-Ciampino city airport. Atmos Environ.

[61] Masiol M, Harrison RM (2015). Quantification of air quality impacts of London Heathrow Airport (UK) from 2005 to 2012. Atmos Environ.

[62] Stettler MEJ, Eastham S, Barrett SRH (2011). Air quality and public health impacts of UK airports. Part I: Emissions. Atmos Environ.

[63] Mokalled T, Le Calvé S, Badaro-Saliba N, Abboud M, Zaarour R, Farah W, Adjizian-Gérard J (2018). Identifying the impact of Beirut Airport’s activities on local air quality - Part I: Emissions inventory of NO2 and VOCs. Atmos Environ.

[64] Rissman J, Arunachalam S, BenDor T, West JJ (2013). Equity and health impacts of aircraft emissions at the Hartsfield-Jackson Atlanta International Airport. Landscape Urban Plan.

[65] Hudda N, Gould T, Hartin K, Larson TV, Fruin SA (2014). Emissions from an international airport increase particle number concentrations 4-fold at 10 km downwind. Environ Sci Technol.

[66] Schlenker W, Walker WR (2015). Airports, Air Pollution, and Contemporaneous Health. Rev Econ Stud.

[67] Pecorari E, Mantovani A, Franceschini C, Bassano D, Palmeri L, Rampazzo G (2016). Analysis of the effects of meteorology on aircraft exhaust dispersion and deposition using a Lagrangian particle model. Sci Total Environ.

[68] Schmid O, Stoeger T (2016). Surface area is the biologically most effective dose metric for acute nanoparticle toxicity in the lung. J Aerosol Sci.

[69] IARC (2010). Diesel and Gasoline Engine Exhausts and Some Nitroarenes. International Agency for Research on Cancer Monographs database.

[70] Salvi S, Blomberg A, Rudell B, Kelly F, Sandström T, Holgate S, Frew A (1999). Acute Inflammatory Responses in the Airways and Peripheral Blood After Short-Term Exposure to Diesel Exhaust in Healthy Human Volunteers. Am J Respir Crit Care Med.

[71] Hashimoto AH, Amanuma K, Hiyoshi K, Sugawara Y, Goto S, Yanagisawa R, Takano H, Masumura K, Nohmi T, Aoki Y (2007). Mutations in the lungs of gpt delta transgenic mice following inhalation of diesel exhaust. Environ Mol Mutagene.

[72] Brightwell J, Fouillet X, Cassano-Zoppi AL, Bernstein D, Crawley F, Duchosal F, Gatz R, Perczel S, Pfeifer H (1989). Tumours of the respiratory tract in rats and hamsters following chronic inhalation of engine exhaust emissions. J Appl Toxicol.

[73] Saber AT, Bornholdt J, Dybdahl M, Sharma AK, Loft S, Vogel U, Wallin H (2005). Tumor necrosis factor is not required for particle-induced genotoxicity and pulmonary inflammation. Arch Toxicol.

[74] Saber AT, Jacobsen NR, Bornholdt J, Kjaer SL, Dybdahl M, Risom L, Loft S, Vogel U, Wallin H (2006). Cytokine expression in mice exposed to diesel exhaust particles by inhalation. Role Tumor Necrosis factor. Particle Fibre Toxicol.

[75] Husain M, Kyjovska ZO, Bourdon-Lacombe J, Saber AT, Jensen KA, Jacobsen NR, Williams A, Wallin H, Halappanavar S, Vogel U (2015). Carbon black nanoparticles induce biphasic gene expression changes associated with inflammatory responses in the lungs of C57BL/6 mice following a single intratracheal instillation. Toxicol Appl Pharmacol.

[76] Saber AT, Jensen KA, Jacobsen NR, Birkedal R, Mikkelsen L, Moller P, Loft S, Wallin H, Vogel U (2012). Inflammatory and genotoxic effects of nanoparticles designed for inclusion in paints and lacquers. Nanotoxicology.

[77] Saber AT, Lamson JS, Jacobsen NR, Ravn-Haren G, Hougaard KS, Nyendi AN, Wahlberg P, Madsen AM, Jackson P, Wallin H (2013). Particle-induced pulmonary acute phase response correlates with neutrophil influx linking inhaled particles and cardiovascular risk. PLoS One.

[78] Jacobsen NR, Moller P, Jensen KA, Vogel U, Ladefoged O, Loft S, Wallin H (2009). Lung inflammation and genotoxicity following pulmonary exposure to nanoparticles in ApoE−/− mice. Particle Fibre Toxicol.

[79] Kyjovska ZO, Jacobsen NR, Saber AT, Bengtson S, Jackson P, Wallin H, Vogel U (2015). DNA strand breaks, acute phase response and inflammation following pulmonary exposure by instillation to the diesel exhaust particle NIST1650b in mice. Mutagenesis.

[80] Vermeulen R, Silverman DT, Garshick E, Vlaanderen J, Portengen L, Steenland K (2014). Exposure-response estimates for diesel engine exhaust and lung cancer mortality based on data from three occupational cohorts. Environ Health Perspect.

[81] Ge C, Peters S, Olsson A, Portengen L, Schüz J, Almansa J, Ahrens W, Bencko V, Benhamou S, Boffetta P (2020). Diesel Engine Exhaust Exposure, Smoking, and Lung Cancer Subtype Risks: A Pooled Exposure-response Analysis of 14 Case-control Studies. Am J Respir Crit Care Med.

[82] IARC. Preamble to the IARC Monographs. January 2019 edn. https://monographs.iarc.fr/iarc-monographs-preamble-preamble-to-the-iarc-monographs/: International Agency for Research in Cancer; 2019.

[83] Childers JW, Witherspoon CL, Smith LB, Pleil JD (2000). Real-time and integrated measurement of potential human exposure to particle-bound polycyclic aromatic hydrocarbons (PAHs) from aircraft exhaust. Environ Health Perspect.

[84] Iavicoli I, Carelli G, Bergamaschi A (2006). Exposure evaluation to airborne polycyclic aromatic hydrocarbons in an italian airport. J Occup Environ Med.

[85] Pirhadi M, Mousavi A, Sowlat MH, Janssen NAH, Cassee FR, Sioutas C (2020). Relative contributions of a major international airport activities and other urban sources to the particle number concentrations (PNCs) at a nearby monitoring site. Environ Pollut.

[86] Buonanno G, Bernabei M, Avino P, Stabile L (2012). Occupational exposure to airborne particles and other pollutants in an aviation base. Environ Pollut.

[87] Møller KL, Thygesen LC, Schipperijn J, Loft S, Bonde JP, Mikkelsen S, Brauer C (2014). Occupational Exposure to Ultrafine Particles among Airport Employees - Combining Personal Monitoring and Global Positioning System. PLOS ONE.

[88] Marie-Desvergne C, Dubosson M, Touri L, Zimmermann E, Gaude-Mome M, Leclerc L, Durand C, Klerlein M, Molinari N, Vachier I (2016). Assessment of nanoparticles and metal exposure of airport workers using exhaled breath condensate. J Breath Res.

[89] Ren J, Liu J, Cao X, Li F, Li J (2017). Ultrafine particles in the cabin of a waiting commercial airliner at Tianjin International Airport, China. Indoor Built Environ.

[90] Ren J, Cao X, Liu J (2018). Impact of atmospheric particulate matter pollutants to IAQ of airport terminal buildings: A first field study at Tianjin Airport, China. Atmos Environ.

[91] Lopes M, Russo A, Monjardino J, Gouveia C, Ferreira F (2019). Monitoring of ultrafine particles in the surrounding urban area of a civilian airport. Atmos Pollut Res.

[92] Marcias G, Casula MF, Uras M, Falqui A, Miozzi E, Sogne E, Pili S, Pilia I, Fabbri D, Meloni F (2019). Occupational Fine/Ultrafine Particles and Noise Exposure in Aircraft Personnel Operating in Airport Taxiway. Environments.

[93] Mokalled T, Adjizian Gérard J, Abboud M, Trocquet C, Nassreddine R, Person V, le Calvé S (2019). VOC tracers from aircraft activities at Beirut Rafic Hariri International Airport. Atmos Pollut Res.

[94] Lin S, Munsie JP, Herdt-Losavio M, Hwang SA, Civerolo K, McGarry K, Gentile TJ (2008). Residential proximity to large airports and potential health impacts in New York State. Int Arch Occup Environ Health.

[95] Senkayi SN, Sattler ML, Rowe N, Chen VCP (2014). Investigation of an association between childhood leukemia incidences and airports in Texas. Atmos Pollut Res.

[96] Penn SL, Boone ST, Harvey BC, Heiger-Bernays W, Tripodis Y, Arunachalam S, Levy JI (2017). Modeling variability in air pollution-related health damages from individual airport emissions. Environ Res.

[97] Henry RC, Mohan S, Yazdani S (2019). Estimating potential air quality impact of airports on children attending the surrounding schools. Atmos Environ.

[98] Møller KL, Brauer C, Mikkelsen S, Loft S, Simonsen EB, Koblauch H, Bern SH, Alkjær T, Hertel O, Becker T (2017). Copenhagen Airport Cohort: air pollution, manual baggage handling and health. BMJ Open.

[99] Møller KL, Brauer C, Mikkelsen S, Bonde JP, Loft S, Helweg-Larsen K, Thygesen LC (2019). Cardiovascular disease and long-term occupational exposure to ultrafine particles: A cohort study of airport workers. Int J Hygiene EnvironHealth.

[100] Lemasters GK, Livingston GK, Lockey JE, Olsen DM, Shukla R, New G, Selevan SG, Yiin JH (1997). Genotoxic changes after low-level solvent and fuel exposure on aircraft maintenance personnel. Mutagenesis.

[101] Tunnicliffe WS, O'Hickey SP, Fletcher TJ, Miles JF, Burge PS, Ayres JG (1999). Pulmonary function and respiratory symptoms in a population of airport workers. Occup Environ Med.

[102] Yang C-Y, Wu T-N, Wu J-J, Ho C-K, Chang P-Y (2003). Adverse Respiratory and Irritant Health Effects in Airport Workers in Taiwan. J Toxicol Environ Health Part A.

[103] Whelan EA, Lawson CC, Grajewski B, Petersen MR, Pinkerton LE, Ward EM, Schnorr TM (2003). Prevalence of respiratory symptoms among female flight attendants and teachers. Occup Environ Med.

[104] Radican L, Blair A, Stewart P, Wartenberg D (2008). Mortality of aircraft maintenance workers exposed to trichloroethylene and other hydrocarbons and chemicals: extended follow-up. J Occup Environ Med.

[105] Erdem O, Sayal A, Eken A, Akay C, Aydin A (2012). Evaluation of genotoxic and oxidative effects in workers exposed to jet propulsion fuel. Int Arch Occup Environ Health.

[106] Visser O, van Wijnen JH, van Leeuwen FE (2005). Incidence of cancer in the area around Amsterdam Airport Schiphol in 1988–2003: a population-based ecological study. BMC Public Health.

[107] Habre R, Zhou H, Eckel SP, Enebish T, Fruin S, Bastain T, Rappaport E, Gilliland F (2018). Short-term effects of airport-associated ultrafine particle exposure on lung function and inflammation in adults with asthma. Environ Int.

[108] Lammers A, Janssen NAH, Boere AJF, Berger M, Longo C, Vijverberg SJH, Neerincx AH, Maitland - van der Zee AH, Cassee FR (2020). Effects of short-term exposures to ultrafine particles near an airport in healthy subjects. Environ Int.

[109] He R-W, Gerlofs-Nijland ME, Boere J, Fokkens P, Leseman D, Janssen NAH, Cassee FR (2020). Comparative toxicity of ultrafine particles around a major airport in human bronchial epithelial (Calu-3) cell model at the air–liquid interface. Toxicol Vitro.

[110] Ferry D, Rolland C, Delhaye D, Barlesi F, Robert P, Bongrand P, Vitte J (2011). Jet exhaust particles alter human dendritic cell maturation. Inflam Res.

[111] Jonsdottir HR, Delaval M, Leni Z, Keller A, Brem BT, Siegerist F, Schönenberger D, Durdina L, Elser M, Burtscher H (2019). Non-volatile particle emissions from aircraft turbine engines at ground-idle induce oxidative stress in bronchial cells. Commun Biol.

[112] Zhou Y, Levy JI (2009). Between-airport heterogeneity in air toxics emissions associated with individual cancer risk thresholds and population risks. Environ Health.

[113] WHO. Ambient (outdoor) air pollution. http://who.int/news-room/fact-sheets/detail/ambient-(outdoor)-air-quality-and-health. Accessed Jan 2021.

[114] Ye RD, Sun L (2015). Emerging functions of serum amyloid A in inflammation. J Leukocyte Biol.

[115] Ye Y, Yue M, Jin X, Chen S, Li Y (2012). The effect of oral tolerance on the roles of small intestinal intraepithelial lymphocytes in murine colitis induced by dextran sodium sulfate. Int J Colorectal Dis.

[116] Yang RB, Mark MR, Gray A, Huang A, Xie MH, Zhang M, Goddard A, Wood WI, Gurney AL, Godowski PJ (1998). Toll-like receptor-2 mediates lipopolysaccharide-induced cellular signalling. Nature.

[117] Stone V, Miller MR, Clift MJD, Elder A, Mills NL, Moller P, Schins RPF, Vogel U, Kreyling WG, Alstrup Jensen K (2017). Nanomaterials Versus Ambient Ultrafine Particles: An Opportunity to Exchange Toxicology Knowledge. Environ Health Perspect.

[118] Carvalho RN, Arukwe A, Ait-Aissa S, Bado-Nilles A, Balzamo S, Baun A, Belkin S, Blaha L, Brion F, Conti D (2014). Mixtures of chemical pollutants at European legislation safety concentrations: how safe are they?. Toxicol Sci.

[119] Naughton SX, Terry AV (2018). Neurotoxicity in acute and repeated organophosphate exposure. Toxicology.

[120] Singh S, Sharma N (2000). Neurological syndromes following organophosphate poisoning. Neurol India.

[121] Howard C, Johnson D, Morton J, Michaelis S, Supplee D, Burdon J (2018). Is a cumulative exposure to a background aerosol of nanoparticles part of the causal mechanism of aerotoxic syndrome.

[122] Castaneda AR, Bein KJ, Smiley-Jewell S, Pinkerton KE (2017). Fine particulate matter (PM2.5) enhances allergic sensitization in BALB/c mice. J Toxicol Env Health Part A.

[123] Inoue KI, Takano H (2011). Aggravating Impact of Nanoparticles on Immune-Mediated Pulmonary Inflammation. Sci World J.

[124] Stone V, Johnston H, Clift MJD (2007). Air pollution, ultrafine and nanoparticle toxicology: Cellular and molecular interactions. IEEE Trans Nanobiosci.

[125] Spira-Cohen A, Chen LC, Kendall M, Lall R, Thurston GD (2011). Personal exposures to traffic-related air pollution and acute respiratory health among Bronx schoolchildren with asthma. Environ Health Perspect.

