# Correction to: A review of health effects associated with exposure to jet engine emissions in and around airports

**DOI:** 10.1186/s12940-021-00705-2

**Published:** 2021-02-24

**Authors:** Katja M. Bendtsen, Elizabeth Bengtsen, Anne T. Saber, Ulla Vogel

**Affiliations:** 1 National Research Centre for the Working Environment, Lersø Parkallé 105, DK-2100 Copenhagen, Denmark; 2 Department of Health Technology, Technical University of Denmark, DK-2800 Kgs Lyngby, Denmark

**Correction to: Environ Health (2021) 20:10**

**https://doi.org/10.1186/s12940-020-00690-y**

Following publication of the original article [1], the authors identified an error in Table 1. The numbers under the column Reference were linked to the References section when it should be linked to the Table footnote.

The original article [1] has been revised
