# Supplementary material for: A review of health effects associated with exposure to jet engine emissions in and around airports
Source: Environ Health. 2021 Feb 6;20:10. doi: 10.1186/s12940-020-00690-y (PMC7866671; doi:10.1186/s12940-020-00690-y)
Supplement: Supplementary file 1 — Additional file 1. [file 12940_2020_690_MOESM1_ESM.docx]

**APPENDIX**

**A. LITERATURE SEARCH**

A number of key papers were identified before the actual search was carried out to be used as guidance for constructing the search strategy (19 papers).

Two literature searches addressing peer reviewed original journal articles were performed in the bibliographic databases, PubMed and the Web of Science Core Collection.

The elements in the search strategy firstly comprised of *who* (crew/personnel, etc.) *AND where* (the setting: airports, etc.) *AND what* (exposure: jet emissions, nanoparticles, etc.) *AND why* (outcome of interest: health effects, occupational safety, etc.). The search profile and result (#44 in the PubMed search shown below) was compared to the key articles. Too many relevant articles were not captured, so it was decided to omit the *who* search set. The final search proved satisfactory (#51 in the PubMed search below)

The search strategy combined sets of keywords using AND/OR terms in Boolean logic. In the first database (PubMed), appropriate index terms and their synonyms were identified in the thesaurus and translated to the Web of Science Core Collection database. Variations in search terms (truncation and abbreviations) and phrase searching is evident from the search profiles below.

The result of the database searches:

PubMed 233 items

Web of Science CC 274 items

Total 507 items

Duplicates 86 items

Unique items 421 items

SEARCH HISTORIES

PubMed

### History

| Search | Query | Items found |
| --- | --- | --- |
| [#51](https://www.ncbi.nlm.nih.gov/pubmed/advanced) | Search **(#23 AND #33 AND #43) AIRPORT AND EXPOSURE AND OUTCOME** | [**233**](https://www.ncbi.nlm.nih.gov/pubmed/?cmd=HistorySearch&querykey=51) |
| [#52](https://www.ncbi.nlm.nih.gov/pubmed/advanced) | Search **(#51 NOT #44)** | [93](https://www.ncbi.nlm.nih.gov/pubmed/?cmd=HistorySearch&querykey=52) |
| [#44](https://www.ncbi.nlm.nih.gov/pubmed/advanced) | Search **(#34 AND #43) CREW/PERSONNEL AND AIRPORT AND EXPOSURE AND OUTCOME** | [140](https://www.ncbi.nlm.nih.gov/pubmed/?cmd=HistorySearch&querykey=44) |

| Search | Query | Items found |
| --- | --- | --- |
| [#44](https://www.ncbi.nlm.nih.gov/pubmed) | Search **(#34 AND #43) CREW/PERSONNEL AND AIRPORT AND EXPOSURE AND OUTCOME** | [140](https://www.ncbi.nlm.nih.gov/pubmed/?cmd=HistorySearch&querykey=44) |
| [#43](https://www.ncbi.nlm.nih.gov/pubmed) | Search **(#35 OR #36 OR #37 OR #38 OR #39 OR #40 OR #41 OR #42) OUTCOME** | [233104](https://www.ncbi.nlm.nih.gov/pubmed/?cmd=HistorySearch&querykey=43) |
| [#42](https://www.ncbi.nlm.nih.gov/pubmed) | Search **"occupational exposure"[Title/Abstract]** | [17110](https://www.ncbi.nlm.nih.gov/pubmed/?cmd=HistorySearch&querykey=42) |
| [#41](https://www.ncbi.nlm.nih.gov/pubmed) | Search **instillation[Title/Abstract]** | [19150](https://www.ncbi.nlm.nih.gov/pubmed/?cmd=HistorySearch&querykey=41) |
| [#40](https://www.ncbi.nlm.nih.gov/pubmed) | Search **("health risk"[Title/Abstract] OR "health risks"[Title/Abstract])** | [35442](https://www.ncbi.nlm.nih.gov/pubmed/?cmd=HistorySearch&querykey=40) |
| [#39](https://www.ncbi.nlm.nih.gov/pubmed) | Search **"occupational health"[Title/Abstract]** | [14443](https://www.ncbi.nlm.nih.gov/pubmed/?cmd=HistorySearch&querykey=39) |
| [#38](https://www.ncbi.nlm.nih.gov/pubmed) | Search **"health effects"[Title/Abstract]** | [25946](https://www.ncbi.nlm.nih.gov/pubmed/?cmd=HistorySearch&querykey=38) |
| [#8](https://www.ncbi.nlm.nih.gov/pubmed) | Search **occupational exposure[MeSH Terms]** | [61606](https://www.ncbi.nlm.nih.gov/pubmed/?cmd=HistorySearch&querykey=8) |
| [#37](https://www.ncbi.nlm.nih.gov/pubmed) | Search **"occupational safety"[Title/Abstract]** | [4571](https://www.ncbi.nlm.nih.gov/pubmed/?cmd=HistorySearch&querykey=37) |
| [#36](https://www.ncbi.nlm.nih.gov/pubmed) | Search **occupational health[MeSH Terms]** | [32307](https://www.ncbi.nlm.nih.gov/pubmed/?cmd=HistorySearch&querykey=36) |
| [#35](https://www.ncbi.nlm.nih.gov/pubmed) | Search **(health[Title/Abstract]) AND (inhale*[Title/Abstract] OR exposure*[Title/Abstract] OR toxicity[Title/Abstract] OR inhalation[Title/Abstract])** | [127424](https://www.ncbi.nlm.nih.gov/pubmed/?cmd=HistorySearch&querykey=35) |
| [#34](https://www.ncbi.nlm.nih.gov/pubmed) | Search **(#24 AND #33)** | [653](https://www.ncbi.nlm.nih.gov/pubmed/?cmd=HistorySearch&querykey=34) |
| [#33](https://www.ncbi.nlm.nih.gov/pubmed) | Search **(#25 OR #26 OR #27 OR #28 OR #29 OR #30 OR #31 OR #32) EXPOSURE** | [2023862](https://www.ncbi.nlm.nih.gov/pubmed/?cmd=HistorySearch&querykey=33) |
| [#32](https://www.ncbi.nlm.nih.gov/pubmed) | Search **((jet[Title/Abstract] OR aircraft*[Title/Abstract])) AND (exhaust[Title/Abstract] OR fumes[Title/Abstract] OR engine*[Title/Abstract] OR emissions[Title/Abstract] OR particles[Title/Abstract] OR "PM"[Title/Abstract] OR "PAH"[Title/Abstract] OR "PAHS"[Title/Abstract] OR "particulate matter"[Title/Abstract])** | [2347](https://www.ncbi.nlm.nih.gov/pubmed/?cmd=HistorySearch&querykey=32) |
| [#31](https://www.ncbi.nlm.nih.gov/pubmed) | Search **"benzoapyrene"[Title/Abstract]** | [6](https://www.ncbi.nlm.nih.gov/pubmed/?cmd=HistorySearch&querykey=31) |
| [#30](https://www.ncbi.nlm.nih.gov/pubmed) | Search **hydrocarbons[MeSH Terms]** | [1786184](https://www.ncbi.nlm.nih.gov/pubmed/?cmd=HistorySearch&querykey=30) |
| [#29](https://www.ncbi.nlm.nih.gov/pubmed) | Search **"PAH"[Title/Abstract]** | [21415](https://www.ncbi.nlm.nih.gov/pubmed/?cmd=HistorySearch&querykey=29) |
| [#28](https://www.ncbi.nlm.nih.gov/pubmed) | Search **polycyclic aromatic hydrocarbons[MeSH Terms]** | [434389](https://www.ncbi.nlm.nih.gov/pubmed/?cmd=HistorySearch&querykey=28) |
| [#27](https://www.ncbi.nlm.nih.gov/pubmed) | Search **nanoparticles[MeSH Terms]** | [119985](https://www.ncbi.nlm.nih.gov/pubmed/?cmd=HistorySearch&querykey=27) |
| [#26](https://www.ncbi.nlm.nih.gov/pubmed) | Search **vehicle emissions[MeSH Terms]** | [9595](https://www.ncbi.nlm.nih.gov/pubmed/?cmd=HistorySearch&querykey=26) |
| [#25](https://www.ncbi.nlm.nih.gov/pubmed) | Search **particulate matter[MeSH Terms]** | [59518](https://www.ncbi.nlm.nih.gov/pubmed/?cmd=HistorySearch&querykey=25) |
| [#24](https://www.ncbi.nlm.nih.gov/pubmed) | Search **(#12 AND #23)** | [8110](https://www.ncbi.nlm.nih.gov/pubmed/?cmd=HistorySearch&querykey=24) |
| [#23](https://www.ncbi.nlm.nih.gov/pubmed) | Search **(#13 OR #14 OR #15 OR #16 OR #17 OR #18 OR #19 OR #20 OR #21 OR #22) AIRPORT** | [40175](https://www.ncbi.nlm.nih.gov/pubmed/?cmd=HistorySearch&querykey=23) |
| [#22](https://www.ncbi.nlm.nih.gov/pubmed) | Search **("air station"[Title/Abstract] OR "air stations"[Title/Abstract])** | [61](https://www.ncbi.nlm.nih.gov/pubmed/?cmd=HistorySearch&querykey=22) |
| [#21](https://www.ncbi.nlm.nih.gov/pubmed) | Search **airplane*[Title/Abstract]** | [1400](https://www.ncbi.nlm.nih.gov/pubmed/?cmd=HistorySearch&querykey=21) |
| [#20](https://www.ncbi.nlm.nih.gov/pubmed) | Search **aircraft*[Title/Abstract]** | [7168](https://www.ncbi.nlm.nih.gov/pubmed/?cmd=HistorySearch&querykey=20) |
| [#19](https://www.ncbi.nlm.nih.gov/pubmed) | Search **(airtraffic[Title/Abstract] OR "air traffic"[Title/Abstract])** | [705](https://www.ncbi.nlm.nih.gov/pubmed/?cmd=HistorySearch&querykey=19) |
| [#18](https://www.ncbi.nlm.nih.gov/pubmed) | Search **aviation*[Title/Abstract]** | [7379](https://www.ncbi.nlm.nih.gov/pubmed/?cmd=HistorySearch&querykey=18) |
| [#17](https://www.ncbi.nlm.nih.gov/pubmed) | Search **airfield*[Title/Abstract]** | [92](https://www.ncbi.nlm.nih.gov/pubmed/?cmd=HistorySearch&querykey=17) |
| [#16](https://www.ncbi.nlm.nih.gov/pubmed) | Search **airport*[Title/Abstract]** | [2498](https://www.ncbi.nlm.nih.gov/pubmed/?cmd=HistorySearch&querykey=16) |
| [#15](https://www.ncbi.nlm.nih.gov/pubmed) | Search **aircraft[MeSH Terms]** | [11124](https://www.ncbi.nlm.nih.gov/pubmed/?cmd=HistorySearch&querykey=15) |
| [#14](https://www.ncbi.nlm.nih.gov/pubmed) | Search **aviation[MeSH Terms]** | [30466](https://www.ncbi.nlm.nih.gov/pubmed/?cmd=HistorySearch&querykey=14) |
| [#13](https://www.ncbi.nlm.nih.gov/pubmed) | Search **airports[MeSH Terms]** | [400](https://www.ncbi.nlm.nih.gov/pubmed/?cmd=HistorySearch&querykey=13) |
| [#12](https://www.ncbi.nlm.nih.gov/pubmed) | Search **(#2 OR #3 OR #4 OR #5 OR #6 OR #7 OR #8 OR #9 OR #10 OR #11) CREW/PERSONNEL** | [1729305](https://www.ncbi.nlm.nih.gov/pubmed/?cmd=HistorySearch&querykey=12) |
| [#11](https://www.ncbi.nlm.nih.gov/pubmed) | Search **personnel[Title/Abstract]** | [75615](https://www.ncbi.nlm.nih.gov/pubmed/?cmd=HistorySearch&querykey=11) |
| [#10](https://www.ncbi.nlm.nih.gov/pubmed) | Search **residents[Title/Abstract]** | [99789](https://www.ncbi.nlm.nih.gov/pubmed/?cmd=HistorySearch&querykey=10) |
| [#9](https://www.ncbi.nlm.nih.gov/pubmed) | Search **passenger*[Title/Abstract]** | [8684](https://www.ncbi.nlm.nih.gov/pubmed/?cmd=HistorySearch&querykey=9) |
| [#7](https://www.ncbi.nlm.nih.gov/pubmed) | Search **(work[Title/Abstract] OR worker[Title/Abstract] OR workers[Title/Abstract] OR working[Title/Abstract])** | [1242464](https://www.ncbi.nlm.nih.gov/pubmed/?cmd=HistorySearch&querykey=7) |
| [#6](https://www.ncbi.nlm.nih.gov/pubmed) | Search **employee*[Title/Abstract]** | [43407](https://www.ncbi.nlm.nih.gov/pubmed/?cmd=HistorySearch&querykey=6) |
| [#5](https://www.ncbi.nlm.nih.gov/pubmed) | Search **volunteer*[Title/Abstract]** | [187457](https://www.ncbi.nlm.nih.gov/pubmed/?cmd=HistorySearch&querykey=5) |
| [#4](https://www.ncbi.nlm.nih.gov/pubmed) | Search **volunteers[MeSH Terms]** | [26341](https://www.ncbi.nlm.nih.gov/pubmed/?cmd=HistorySearch&querykey=4) |
| [#3](https://www.ncbi.nlm.nih.gov/pubmed) | Search **staff[Title/Abstract]** | [143354](https://www.ncbi.nlm.nih.gov/pubmed/?cmd=HistorySearch&querykey=3) |
| [#2](https://www.ncbi.nlm.nih.gov/pubmed) | Search **crew[Title/Abstract]** | [3964](https://www.ncbi.nlm.nih.gov/pubmed/?cmd=HistorySearch&querykey=2) |

-----------------------------------------------------------------------------------------------------------------------------------------------

Web of Science Core Collection

| Search History | | | | | |
| --- | --- | --- | --- | --- | --- |
| **Set** | **Results** |  |  |  |  |
|  | | | | | |
| # 9 | [**274**](http://apps.webofknowledge.com/summary.do?product=WOS&doc=1&qid=13&SID=E5ibaoMhTRwhRNflPse&search_mode=CombineSearches&update_back2search_link_param=yes) | #8 AND #5  *Indexes=SCI-EXPANDED, SSCI, A&HCI Timespan=1900-2019* |  |  |  |
|  | | | | | |
| # 8 | [**269,993**](http://apps.webofknowledge.com/summary.do?product=WOS&doc=1&qid=12&SID=E5ibaoMhTRwhRNflPse&search_mode=CombineSearches&update_back2search_link_param=yes) | #7 OR #6  *Indexes=SCI-EXPANDED, SSCI, A&HCI Timespan=1900-2019* |  |  |  |
|  | | | | | |
| # 7 | [**185,282**](http://apps.webofknowledge.com/summary.do?product=WOS&doc=1&qid=11&SID=E5ibaoMhTRwhRNflPse&search_mode=GeneralSearch&update_back2search_link_param=yes) | **TOPIC:** (health) *AND* **TOPIC:** (inhale* OR exposure* OR expose* OR toxicity OR inhalation OR genotoxic*)  *Indexes=SCI-EXPANDED, SSCI, A&HCI Timespan=1900-2019* |  |  |  |
|  | | | | | |
| # 6 | [**124,958**](http://apps.webofknowledge.com/summary.do?product=WOS&doc=1&qid=10&SID=E5ibaoMhTRwhRNflPse&search_mode=GeneralSearch&update_back2search_link_param=yes) | **TOPIC:** ("occupational health") *OR* **TOPIC:** ("occupational safety") *OR* **TOPIC:** ("occupational exposure") *OR* **TOPIC:** ("health effects") *OR* **TOPIC:** ("health risk" OR "health risks") *OR* **TOPIC:** (instillation) *OR* **TOPIC:** ("workers exposed")  *Indexes=SCI-EXPANDED, SSCI, A&HCI Timespan=1900-2019* |  |  |  |
|  | | | | | |
| # 5 | [**16,039**](http://apps.webofknowledge.com/summary.do?product=WOS&doc=1&qid=9&SID=E5ibaoMhTRwhRNflPse&search_mode=CombineSearches&update_back2search_link_param=yes) | #4 AND #1  *Indexes=SCI-EXPANDED, SSCI, A&HCI Timespan=1900-2019* |  |  |  |
|  | | | | | |
| # 4 | [**946,679**](http://apps.webofknowledge.com/summary.do?product=WOS&doc=1&qid=8&SID=E5ibaoMhTRwhRNflPse&search_mode=CombineSearches&update_back2search_link_param=yes) | #3 OR #2  *Indexes=SCI-EXPANDED, SSCI, A&HCI Timespan=1900-2019* |  |  |  |
|  | | | | | |
| # 3 | [**57,690**](http://apps.webofknowledge.com/summary.do?product=WOS&doc=1&qid=7&SID=E5ibaoMhTRwhRNflPse&search_mode=GeneralSearch&update_back2search_link_param=yes) | **TOPIC:** (jet OR aviation OR aircraft*) *AND* **TOPIC:** (exhaust OR fumes OR engine* OR emissions OR particles OR "PM" OR "PAH*" OR "particulate matter")  *Indexes=SCI-EXPANDED, SSCI, A&HCI Timespan=1900-2019* |  |  |  |
|  | | | | | |
| # 2 | [**892,373**](http://apps.webofknowledge.com/summary.do?product=WOS&doc=1&qid=4&SID=E5ibaoMhTRwhRNflPse&search_mode=GeneralSearch&update_back2search_link_param=yes) | **TOPIC:** ("particulate matter") *OR* **TOPIC:** ("vehicle emissions") *OR* **TOPIC:** (nanoparticles) *OR* **TOPIC:** ("polycyclic aromatic hydrocarbons") *OR* **TOPIC:** ("PAH" OR "PAHs") *OR* **TOPIC:** (hydrocarbons) *OR* **TOPIC:** (benzoapyrene)  *Indexes=SCI-EXPANDED, SSCI, A&HCI Timespan=1900-2019* |  |  |  |
|  | | | | | |
| # 1 | [**85,056**](http://apps.webofknowledge.com/summary.do?product=WOS&doc=1&qid=1&SID=E5ibaoMhTRwhRNflPse&search_mode=GeneralSearch&update_back2search_link_param=yes) | **TOPIC:** (airport*) *OR* **TOPIC:** (aviation*) *OR* **TOPIC:** (aircraft*) *OR* **TOPIC:** (airtraffic OR "air traffic") *OR* **TOPIC:** (airplane) *OR* **TOPIC:** ("air station*")  *Indexes=SCI-EXPANDED, SSCI, A&HCI Timespan=1900-2019/* |  |  |  |

**B. SELECTION STRATEGY**

Our aim was to summarize the available studies on health effects of airport emissions with main focus on particles and on occupational exposure. We report findings of in vitro and in vivo studies, describe studies of residents near airports comprising volunteers and correlation/regression models using health/epidemiological data in combination with meteorological and/or environmental data, and present the body of available studies on occupational exposure and direct toxicological evaluations in airport workers.

We initially examined the scientific literature by consulting the reference list of reviews and relevant studies (snowball method), expanding our knowledge and establishing a solid bibliography. To secure a wide representation of study types and seek to include all relevant studies on direct health assessments of airport emissions with main focus on particles, we conducted a systematic search in two databases.

The final search resulted in 421 unique papers, which were compiled with title and complete abstracts. Papers of clear irrelevance were discarded based on titles and papers of remote relevance were filed. Relevant papers were included in the review, as direct sources within the specific sections or used in the introduction or for perspectives.
